# Genomic Surveillance Reveals Emergence and Spread of Macrolide-Resistant *Mycoplasma pneumoniae* in Australia During the 2023–2024 Epidemic

**DOI:** 10.1093/infdis/jiag163

**Published:** 2026-03-21

**Authors:** Kingsley King-Gee Tam, Carl J E Suster, Winkie Fong, Tanya Golubchik, Varsha Sivalingam, Neisha Jeoffreys, Enoch Tay, Danny Ko, Michael C Wehrhahn, Andrew N Ginn, Jennifer Robson, Indya Gardner, Lito E Papanicolas, Karina Kennedy, Maryza Graham, Thomas Tran, David Speers, Louise Cooley, Rob W Baird, Ella M Meumann, Jaimee Harbidge, Stuart Campbell, Kerri Basile, Sharon C A Chen, Vitali Sintchenko, Jen Kok, Rebecca J Rockett

**Affiliations:** Centre for Infectious Diseases and Microbiology–Public Health, Westmead Hospital, Westmead, New South Wales, Australia; School of Medical Sciences, Faculty of Medicine and Health, Sydney Infectious Diseases Institute, The University of Sydney, Westmead, New South Wales, Australia; Centre for Infectious Diseases and Microbiology–Public Health, Westmead Hospital, Westmead, New South Wales, Australia; School of Medical Sciences, Faculty of Medicine and Health, Sydney Infectious Diseases Institute, The University of Sydney, Westmead, New South Wales, Australia; Centre for Infectious Diseases and Microbiology–Public Health, Westmead Hospital, Westmead, New South Wales, Australia; School of Medical Sciences, Faculty of Medicine and Health, Sydney Infectious Diseases Institute, The University of Sydney, Westmead, New South Wales, Australia; Centre for Infectious Diseases and Microbiology–Public Health, Westmead Hospital, Westmead, New South Wales, Australia; School of Medical Sciences, Faculty of Medicine and Health, Sydney Infectious Diseases Institute, The University of Sydney, Westmead, New South Wales, Australia; Centre for Infectious Diseases and Microbiology–Public Health, Westmead Hospital, Westmead, New South Wales, Australia; Centre for Infectious Diseases and Microbiology Laboratory Services, NSW Health Pathology, Institute of Clinical Pathology and Medical Research, Westmead Hospital, Westmead, New South Wales, Australia; Centre for Infectious Diseases and Microbiology Laboratory Services, NSW Health Pathology, Institute of Clinical Pathology and Medical Research, Westmead Hospital, Westmead, New South Wales, Australia; Centre for Infectious Diseases and Microbiology Laboratory Services, NSW Health Pathology, Institute of Clinical Pathology and Medical Research, Westmead Hospital, Westmead, New South Wales, Australia; Centre for Infectious Diseases and Microbiology Laboratory Services, NSW Health Pathology, Institute of Clinical Pathology and Medical Research, Westmead Hospital, Westmead, New South Wales, Australia; Douglass Hanly Moir Pathology, A Sonic Healthcare Practice, Macquarie Park, New South Wales, Australia; Douglass Hanly Moir Pathology, A Sonic Healthcare Practice, Macquarie Park, New South Wales, Australia; Sullivan Nicolaides Pathology, A Sonic Healthcare Practice, Bowen Hills, Queensland, Australia; Sullivan Nicolaides Pathology, A Sonic Healthcare Practice, Bowen Hills, Queensland, Australia; SA Pathology, Adelaide, South Australia, Australia; ACT Pathology, Woden, Australian Capital Territory, Australia; Victorian Infectious Diseases Reference Laboratory, Royal Melbourne Hospital, Doherty Institute for Infection and Immunity, Melbourne, Victoria, Australia; Department of Infectious Diseases, University of Melbourne, Doherty Institute for Infection and Immunity, Melbourne, Victoria, Australia; Department of Microbiology, Monash Health, Clayton, Victoria, Australia; Monash Infectious Diseases, Monash Health, Clayton, Victoria, Australia; Faculty of Medicine, Nursing and Health Sciences, Monash University, Clayton, Victoria, Australia; Victorian Infectious Diseases Reference Laboratory, Royal Melbourne Hospital, Doherty Institute for Infection and Immunity, Melbourne, Victoria, Australia; Department of Microbiology, PathWest Laboratory Medicine WA, Nedlands, Western Australia, Australia; Department of Microbiology and Infectious Diseases, Royal Hobart Hospital, Hobart, Tasmania, Australia; Territory Pathology, Royal Darwin Hospital, Casuarina, Northern Territory, Australia; Territory Pathology, Royal Darwin Hospital, Casuarina, Northern Territory, Australia; Global and Tropical Health Division, Menzies School of Health Research, Casuarina, Northern Territory, Australia; Territory Pathology, Royal Darwin Hospital, Casuarina, Northern Territory, Australia; Territory Pathology, Royal Darwin Hospital, Casuarina, Northern Territory, Australia; Centre for Infectious Diseases and Microbiology–Public Health, Westmead Hospital, Westmead, New South Wales, Australia; School of Medical Sciences, Faculty of Medicine and Health, Sydney Infectious Diseases Institute, The University of Sydney, Westmead, New South Wales, Australia; Centre for Infectious Diseases and Microbiology Laboratory Services, NSW Health Pathology, Institute of Clinical Pathology and Medical Research, Westmead Hospital, Westmead, New South Wales, Australia; Centre for Infectious Diseases and Microbiology–Public Health, Westmead Hospital, Westmead, New South Wales, Australia; School of Medical Sciences, Faculty of Medicine and Health, Sydney Infectious Diseases Institute, The University of Sydney, Westmead, New South Wales, Australia; Centre for Infectious Diseases and Microbiology Laboratory Services, NSW Health Pathology, Institute of Clinical Pathology and Medical Research, Westmead Hospital, Westmead, New South Wales, Australia; Centre for Infectious Diseases and Microbiology–Public Health, Westmead Hospital, Westmead, New South Wales, Australia; School of Medical Sciences, Faculty of Medicine and Health, Sydney Infectious Diseases Institute, The University of Sydney, Westmead, New South Wales, Australia; Centre for Infectious Diseases and Microbiology Laboratory Services, NSW Health Pathology, Institute of Clinical Pathology and Medical Research, Westmead Hospital, Westmead, New South Wales, Australia; Centre for Infectious Diseases and Microbiology–Public Health, Westmead Hospital, Westmead, New South Wales, Australia; School of Medical Sciences, Faculty of Medicine and Health, Sydney Infectious Diseases Institute, The University of Sydney, Westmead, New South Wales, Australia; Centre for Infectious Diseases and Microbiology Laboratory Services, NSW Health Pathology, Institute of Clinical Pathology and Medical Research, Westmead Hospital, Westmead, New South Wales, Australia; Centre for Infectious Diseases and Microbiology–Public Health, Westmead Hospital, Westmead, New South Wales, Australia; School of Medical Sciences, Faculty of Medicine and Health, Sydney Infectious Diseases Institute, The University of Sydney, Westmead, New South Wales, Australia

**Keywords:** *Mycoplasma pneumoniae*, macrolide resistance, whole-genome sequencing, targeted metagenomic sequencing, antimicrobial stewardship, Australia

## Abstract

**Background:**

The resurgence of *Mycoplasma pneumoniae*, first reported in China in 2023, was attributed to waning postpandemic immunity with increases in macrolide-resistant *M. pneumoniae* (MRMP) (> 80%). In Australia, infections peaked in early 2024, particularly among children under 15 years. While MRMP remains low in Europe, North America, and Australia (< 5%), limited routine testing restricts understanding of resistance dynamics. As macrolides are first-line therapy in many settings, MRMP surveillance is essential for guiding empirical treatment.

**Methods:**

We applied a capture-based targeted metagenomic sequencing (targeted next-generation sequencing [tNGS]) to polymerase chain reaction (PCR)-positive *M. pneumoniae* specimens (n = 356) from across Australia. This approach enabled whole-genome recovery and MRMP detection directly from clinical specimens, without culture. MRMP detections were benchmarked against PCR and data were analyzed to assess associations between resistance and health care utilization.

**Results:**

This is the first genomics-informed national study of *M. pneumoniae* in Australia. We recovered 124 high-quality genomes, revealing a genetically diverse population with cocirculation of P1 type 1 (69%) and type 2 (31%). MRMP was identified in 13% of genomes, all belonging to clades that prior to 2024 had only been reported in Asia (ST3 and ST14). MRMP cases were geographically widespread, suggesting importation and local transmission. Unlike reports from China, macrolide-susceptible clades (ST3, ST7, ST17, and ST20) predominated (87%) and were associated with significantly lower health care utilization compared to MRMP cases.

**Conclusions:**

Our findings demonstrate the utility of tNGS for genomic epidemiology and highlight the need for MRMP surveillance. Although macrolides remain effective in Australia, emerging MRMP strains require close monitoring to inform treatment guidelines and antimicrobial stewardship.

## Teaser

Targeted metagenomic sequencing enabled national genomic surveillance of *Mycoplasma pneumoniae* in Australia, revealing a genetically diverse, predominantly macrolide-sensitive population during the 2023–2024 epidemic, with macrolide-resistant strains restricted to clades previously only reported in Asia.

## Graphical Abstract

**Graphical Abstract:**
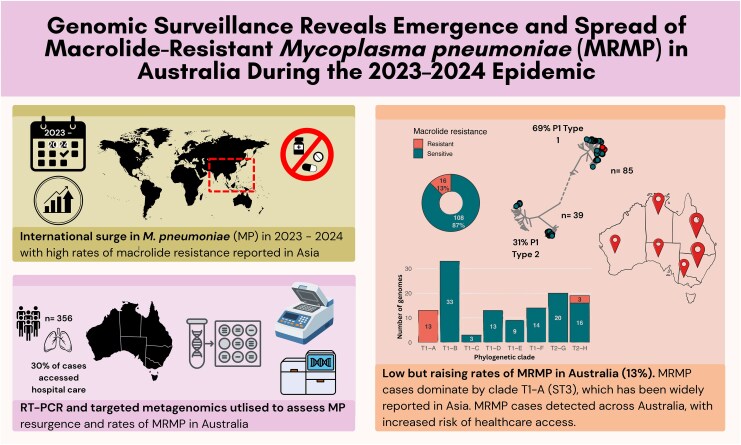

Lower respiratory tract infections remain a leading cause of age-standardized mortality [1]. *Mycoplasma pneumoniae*, one of the smallest free-living bacterial pathogens, accounts for 4%–8% of community-acquired pneumonia case and can contribute up to 40% of cases during epidemic years [2]. Humans are the only known hosts, and *M. pneumoniae* most often causes mild respiratory infections, although children > 5 years are more likely to be infected [3–5]. Epidemic peaks of *M. pneumoniae* occur every 3–7 years, with the last significant outbreak prior to the coronavirus disease 2019 (COVID-19) pandemic in 2020 [6]. In late 2023, a surge in pediatric pneumonia in northern China was largely attributed to the reemergence of *M. pneumoniae* following the easing of COVID-19–related nonpharmaceutical interventions, including travel restrictions [7, 8].

Increases in respiratory disease caused by *M. pneumoniae* were observed globally in 2024 [7, 9–12]. In Australia during early 2024, a 2–6-fold increase over the 5-year average in emergency department presentations for pneumonia was noted, coinciding with increased *M. pneumoniae* circulation [13]. *M. pneumoniae* cases in the 5–16-year age group were overrepresented in these presentations, indicating it causes a significant health burden in pediatric populations.

*M. pneumoniae* has a genetically stable 0.81 million base pair (bp) genome. The bacterium lacks a cell wall; therefore, it is resistant to β-lactam antibiotics but susceptible to antibiotics that disrupt DNA replication (fluoroquinolones) or protein synthesis (macrolides or tetracycline). Given pediatric safety concerns with fluoroquinolones, empirical respiratory infection treatment guidelines generally rely on macrolides [2]. Macrolide-resistant *M. pneumoniae* (MRMP) was identified as early as 1970, but circulation in the community was not reported until the early 2000s [14–16]. Resistance is linked to point mutations in the peptidyl transferase V domain of the 23S rRNA region, most commonly A2063G; however, other mutations within the region and in ribosomal proteins L4 and L22 have also been reported [17–19]. The incidence of MRMP is geographically distinct [20] with resistance detected in < 10% of *M. pneumoniae* cases in Europe [10, 11, 21], North America [22, 23], and Oceania [24–26], but in 80%–90% of cases in Asia [7, 9, 14, 27–32].

Diversification of the primary antigen of *M. pneumoniae,* the P1 adhesin protein, has also been proposed as a driver of epidemic peaks [21, 30]. Two main P1 adhesin types, type 1 (P1-T1) and type 2 (P1-T2), have been described via sequencing of the P1 adhesin repetitive regions (RepMP2/3 and RepMP4). These repetitive regions are present at 18 genomic sites throughout the *M. pneumoniae* genome, which allow diversification through homologous recombination [33]. However, the limited use and comparability of detection and characterization methods has restricted the investigation of *M. pneumoniae* molecular epidemiology. In addition, with limited capacity to culture *M. pneumoniae* in diagnostic laboratories, sequencing studies based on Sanger sequencing of the P1 adhesin gene and multilocus sequence typing or shotgun metagenomics have been used, but comprehensive data remain sparse, particularly in regions reporting low rates of MRMP prevalence.

This study aimed to utilize novel targeted metagenomics (targeted next-generation sequencing [tNGS]) methods to investigate the genomic diversity of circulating *M. pneumoniae* in Australia, particularly during the 2023–2024 resurgence. Genomic data were used to determine the prevalence of MRMP infections, including but not limited to the A2063G mutation in the 23S rRNA region. We hypothesized that increasing MRMP may have contributed to the high circulation of *M. pneumoniae* in Australia during the 2023–2024 epidemic and should be monitored to inform empirical treatment guidelines for community-acquired pneumonia.

## METHODS

*M. pneumoniae* is not a notifiable infectious disease in Australia. To quantify the surge in *M. pneumoniae* cases in Australia, weekly *M. pneumoniae* polymerase chain reaction (PCR) positivity data were supplied by 2 large, private pathology providers, Douglass Hanly Moir Pathology and Sullivan Nicolaides Pathology (Sonic HealthCare Limited, Australia) between 1 January 2018 and 7 October 2024. Further study methods, including extended details of the sample cohort, library preparation, probe capture methodology, bioinformatic, and statistical methods, can be found in the Supplementary Methods, Supplementary Data 3.

### Sample Cohort

**Figure 1.**
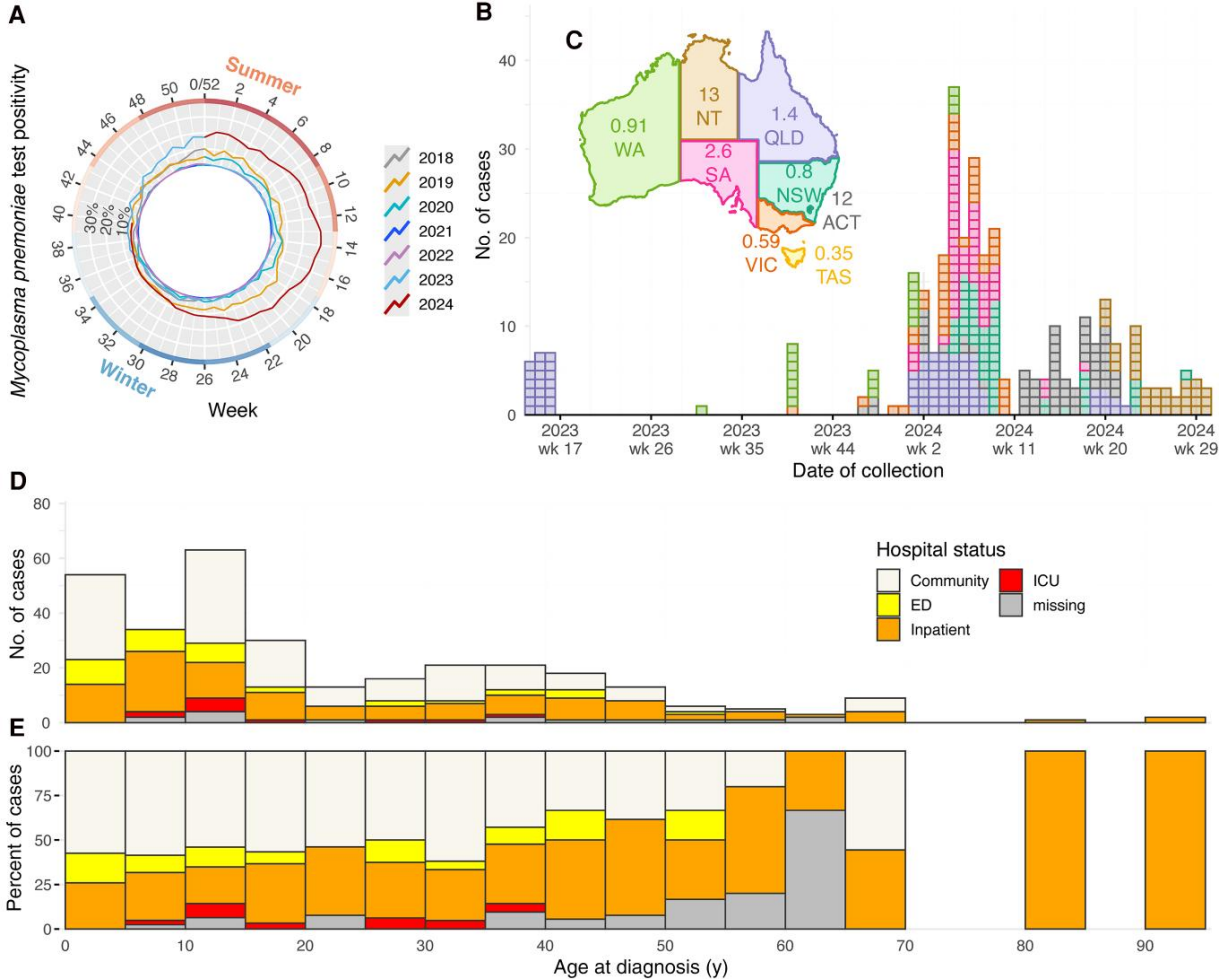
Spatial temporal distribution and demographic information of *Mycoplasma pneumoniae* cases in Australia included in the study. *A*, PCR positivity for *M. pneumoniae* remained less than 10% between 1 January 2018 and 1 December 2023. From the start of 2024, *M. pneumoniae* was detected in over 20% of specimens tested, peaking in week 14, 2024 with a PCR positivity of 32%. Positivity rates for *M. pneumoniae* remained greater than 10% until week 28 in mid-2024. The color bars on the outside of the radial graph indicate summer and winter seasons in Australia. Line colors indicate the year of PCR positivity. *B*, Epidemic curve of *M. pneumoniae* cases collected as part of this study between week 17, 2023 and week 29, 2024. *C*, State-level distribution of *M. pneumoniae* cases, adjusted to a rate of 100 000 individuals per state or territory population. *D*, *M. pneumoniae* case counts per age category overlayed with associated health care access, where white indicates cases managed in the community, yellow highlights cases presenting to a hospital ED with or without admission, orange highlights cases that were admitted to a hospital, red indicates cases admitted to an ICU, and grey highlights cases where health care access was unknown. *E*, Health care access as a proportion of total case counts for each age category. Abbreviations: ACT, Australian Capital Territory; ED, emergency department; ICU, intensive care unit; NSW, New South Wales; NT, Northern Territory; PCR, polymerase chain reaction; QLD, Queensland; SA, South Australia; TAS, Tasmania; VIC, Victoria; WA, Western Australia.

A total of 356 *M. pneumoniae* PCR-positive respiratory tract specimens, collected from 352 patients, were referred from public and private diagnostic laboratories in all jurisdictions of Australia (Figure 1). The study was approved by the Western Sydney Local Health District Human Research Ethics and Governance Committee (project identifier, 2019/PID14240).

The cases were divided into historical cases (n = 31, with sample collection between 1 September 2014 and 1 July 2021) and cases identified during the 2023–2024 epidemic period (n = 325, 3 April 2023 to 22 July 2024) (Figure 1*B* and *C*). Demographic information was collected including age at diagnosis and severity of illness based on health care seeking behavior.

### Library Preparation

Briefly 5 μL of total nucleic acid was used to prepare libraries using the Library Preparation Enzymatic Fragmentation kit 1.0 (Twist Biosciences). Libraries were dried down prior to overnight (17 hour) hybridization. A custom bait panel was generated to capture the entire *M. pneumoniae* pangenome (*M. pneumoniae* panel) Samples were sequenced using Illumina chemistry resulting in 150-bp paired-end reads with the aim of generating 1–2 million reads per sample.

### Bioinformatic Analysis

Sequencing reads were trimmed using Trimmomatic and classified by Kraken2. Trimmed reads were mapped to NCBI RefSeq assembly CP003913.2 using Snippy (version 4.6.0) [34]. Reference-based mapping quality was assessed using samtools (version 1.10) and only *M. pneumoniae* genomes with > 80% genome coverage and a minimum of 12 × depth were included. A full genome alignment was generated using Snippycore (version 4.1.0) and recombination was removed using Gubbins (version 2.3.4) [35]. Single-nucleotide polymorphism (SNP) sites were used to determine core SNP differences between genomes. Maximum-likelihood phylogenetic reconstruction was performed with automated model selection as implemented in IQ-TREE [36] (version 1.6.7) and 1000 bootstrap replicates with zero-length branches collapsed.

To estimate the timing of divergence events, we applied BactDating (version 1.1) [37]. The midpoint-rooted phylogeny constructed for both P1-T1 and P1-T2 was used with branch lengths rescaled to the alignment length (3484 bp P1-T1 and 1848 bp P1-T2).

Macrolide resistance was detected in silico from variant calling at 23S rRNA positions (nucleotide positions 2017, 2054, 2058, 2063, 2064, 2071, 2353, and 2617) [19, 38]. Additional resistance markers were investigated including tetracycline resistance mutations in the 16S rRNA (G1193A, T968C), and quinolone resistance by mutations in *gyrA*, *gyrB*, and *parC* [38].

### Macrolide Resistance by RT-PCR

All clinical specimens were subjected to a modified *M. pneumoniae* 23S rRNA resistance real-time PCR assay targeting mutations at 2063 and 2064 loci [39]. Fluorescent signal acquisition was conducted at the end of every cycle during the amplification stage and cycle threshold (Ct) was determined by Rotor-Gene Q (Qiagen) software. Specimens with ΔCt (Ct_YAK_ − Ct_FAM_) that exceeded 1.5 were interpreted as MRMP.

### Statistical Analysis

Genome read depth and genome coverage were compared between freshly reextracted original samples and referred DNA extracts using a Kruskal-Wallis rank sum test. To assess statistical association between MRMP and health care access, a Pearson χ^2^ test with Yates continuity correction was performed. To assess the association between phylogenetic group and health care access, an age-adjusted logistic regression was used. Statistical significance was defined as a 95% CI for the odds ratio that did not include 1. Statistical analysis and visualization were performed using R (version 4.2.2).

## RESULTS

### Resurgence of *M. pneumoniae* in Australia in 2023 and 2024

*M. pneumoniae* PCR positivity between 2018 and 2022 was less than 10% (Figure 1*A*). From late 2023 (epidemiological week 47) PCR positivity surpassed 10%, reaching 21% by the first week of 2024 and peaking at 31% by week 14, 2024. *M. pneumoniae* detection rate remained > 10% until mid-2024 (week 27, 2024). Further study results including extended details of the genomics analysis, antimicrobial resistance detection, assessment of health care utilization, phylogenetic analysis, and RT-PCR macrolide resistance detection can be found in the Supplementary Results, Supplementary Data 3.

Demographics of the cohort indicated 44% (157/357) of *M. pneumoniae* cases were diagnosed in children less than 15 years of age (Figure 1*D* and 1*E*). Just over half of *M. pneumoniae* cases (52%, 186/352) in the study were managed in the community; however, 44% required hospital presentation, comprising emergency department presentation 10% (35/352), hospital admission 31% (109/352), and ICU admission 3% (10/352). Health care seeking behavior was not available for 5% (17/352) of *M. pneumoniae* cases (Figure 1*E*).

### Genomic Analysis

**Figure 2.**
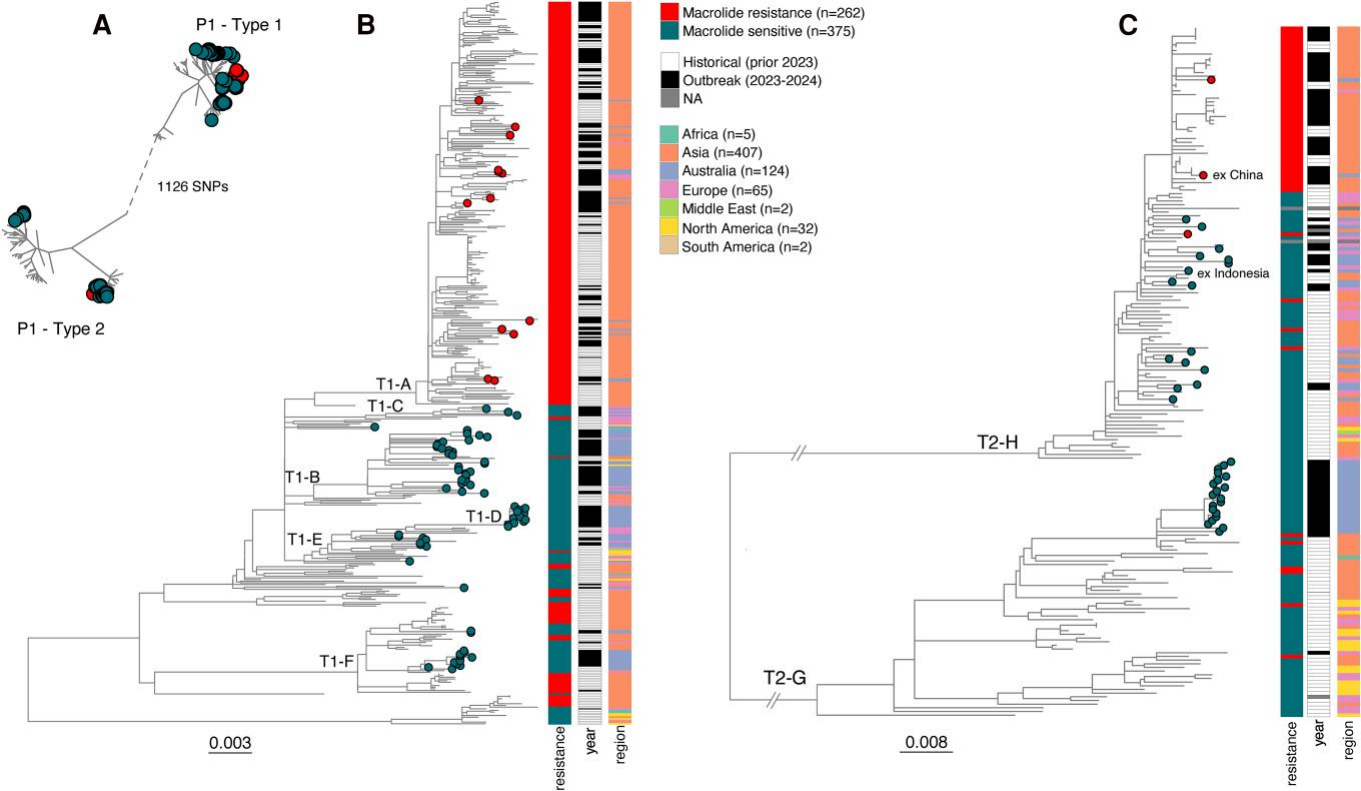
Phylogenetic analysis of international and Australian *Mycoplasma pneumoniae* genomes. *A*, Unrooted phylogeny of 637 *M. pneumoniae* genomes demonstrating deep bifurcation into P1 adhesin types 1 and 2. Australian genomes generated for this study are highlighted by colored circle tips in all 3 phylogenies (n = 124): genomes containing mutations conferring macrolide resistance are highlighted in red (n = 16) and macrolide-sensitive genomes in green (n = 108). Maximum likelihood phylogenetic analysis was performed for P1 adhesion type 1 (*B*) and type 2 (*C*). The left meta bar next to each phylogeny indicates genomes that contain mutations conferring macrolide resistance highlighted in red (n = 262) and susceptible genomes in green (n = 375). The middle metabar indicates *M. pneumoniae* genomes collected from cases prior to 2023 in white (n = 391) and attributed to the 2023–2024 epidemic in black (n = 241); year of collection was not available for 4 international genomes highlighted in grey. The right metabar indicates the geographical region of collection; more than half of available *M. pneumoniae* genomes were collected from the Asian region (orange, 64%, n = 407/637) with poor geographical representation of *M. pneumoniae* genomes collected from Europe (purple, 10%, 65/637), North America (yellow, 5%, 32/637), South America (light brown, 0.3%, 2/637), the Middle East (green, 0.3%, 2/637), and Africa (dark green, 0.8%, 5/637). Abbreviations: NA, not available; SNP, single-nucleotide polymorphism.

tNGS was attempted on 322 specimens; this resulted in the recovery of 124 complete *M. pneumoniae* genomes using tNGS (40%, 124/322) (Figure 2*A*; sequence read archive [SRA] reference number provided in Supplementary Data 2). Of the complete genomes, 15 cases were from the historical *M. pneumoniae* cohort (collected between 2014 and 2021) and 109 from the recent epidemic (2023–2024). Successful genome recovery was associated with lower *M. pneumoniae* PCR Ct values, where specimens with Ct ≤ 20 had a whole *M. pneumoniae* genome recovery rate of 94% (16/17). Despite whole genomes being successfully recovered in *M. pneumoniae* specimens with low bacterial load (highest Ct value, 32.07), lower genome recovery rates were observed with increasing Ct value ranges: 20 to < 25 (53%), 25 to < 30 (28%), and 30 to < 35 (14%) (Supplementary Data 3, Supplementary Figure 1). High-quality genomes were recovered with an average depth of 121.5 × (range 13–2579 ×) and genome coverage of 99.81% (range 99.2%–100%) (Supplementary Data 2 and 3, Supplementary Figure 2).

The *M. pneumoniae* phylogeny was constructed including 512 international *M. pneumoniae* genomes (Supplementary Data 1), and demonstrated deep divergence into 2 primary branches containing study genomes classified as P1-T1 (69%, 85/124) or P1-T2 (31%, 39/124). Both P1 adhesin types cocirculated during 2023–2024; however, P1-T1 dominated (Figure 2*A*). A separate phylogenetic analysis was conducted for each P1 type. P1-T1 genomes had a high degree of genetic diversity, falling into 6 of 9 major clades: T1-A (ST3, n = 13), T1-B (ST3, n = 33), T1-C (ST-, n = 3), T1-D (ST20, n = 13), T1-E (ST20, n = 9), and T1-F (ST17, n = 14) (Figure 2*B*). Phylogenetic analysis of P1-T2 genomes demonstrated that Australian cases were clustered into 2 of 4 major clades: T2-G (ST7, n = 20) and T2-H (ST14, n = 19) (Figure 2*C*).

### Genomics-Based Resistance Assessment

**Figure 3.**
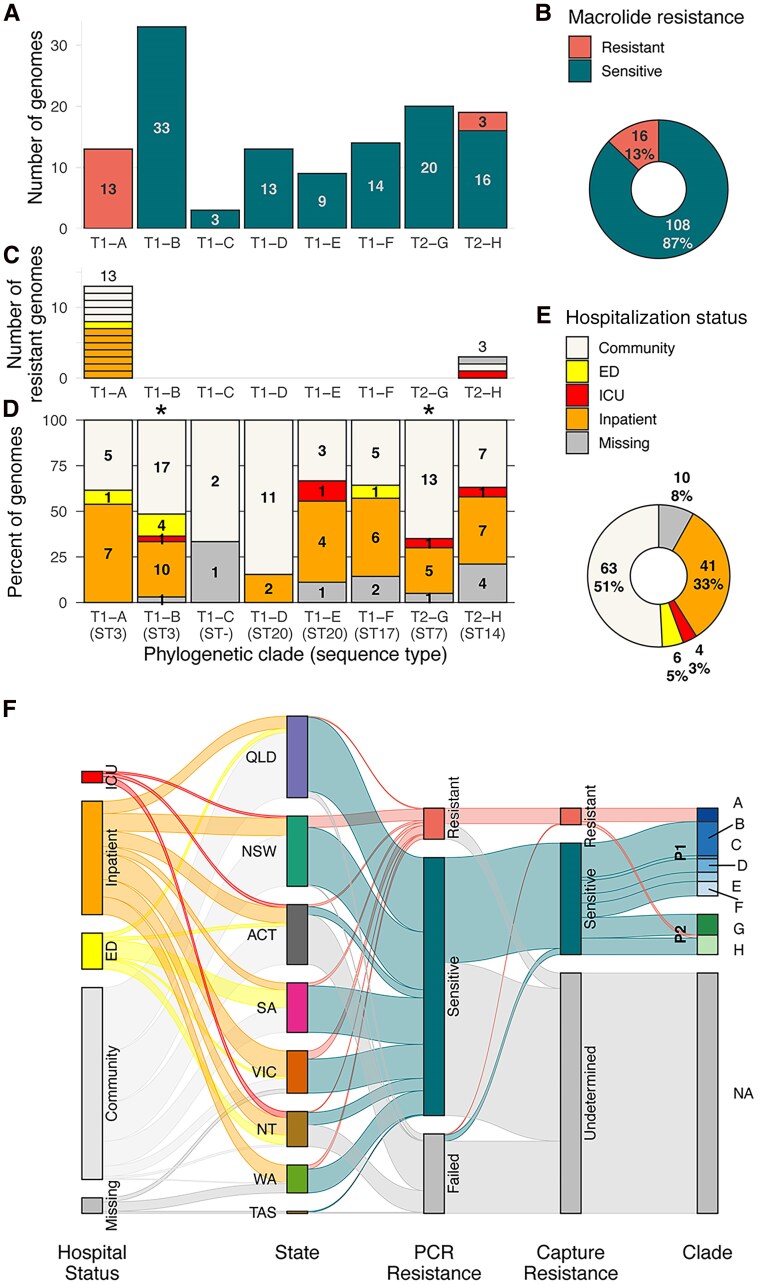
Phylogenetic classification of *Mycoplasma pneumoniae* cases in this study. *A,* The *M. pneumoniae* genomes in this study can be classified into 8 phylogenetic clades, 6 P1-T1 clades, T1-A (n = 13), T1-B (n = 33), T1-C (n = 3), T1-D (n = 13), T1-E (n = 9), and T1-F (n = 14); and 2 P1-T2 clades, T2-G (n = 20) and T2-H (n = 19). Macrolide-resistant *M. pneumoniae* was only detected in clade T1-A (n = 13/13) and T2-H (n = 3/19), representing 13% of all *M. pneumoniae* genomes in this study (*B*). *C*, No statistically significant increase in health care access was detected between macrolide-resistant and -sensitive *M. pneumoniae* when comparing macrolide-resistant *M. pneumoniae* cases identified using genomics*. D*, Health care access reported for *M. pneumoniae* cases was also compared across phylogenetic clades with a significantly lower age-adjusted OR of health care access reported for clades T1-B (OR, 0.092; 95% CI, .011–.54; *P* = .0142) and T2-G (OR, 0.20; 95% CI, .04–.91; 4*P* = .0426) indicated with an asterisk. *E*, Hospital care (ED, inpatient, or ICU admission) was required for 41% of *M. pneumoniae* cases where a complete *M. pneumoniae* genome could be recovered. *F*, A Sankey plot was constructed to summaries the geographical distribution, health care access requirements, and macrolide-resistant *M. pneumoniae* case distribution detected by both genomic and PCR methods in our study. Although only 13% of cases were macrolide resistant, these cases were distributed across all but 1 Australian state. Abbreviations: ACT, Australian Capital Territory; ED, emergency department; ICU, intensive care unit; NA, not available; NSW, New South Wales; NT, Northern Territory; OR, odds ratio; PCR, polymerase chain reaction; QLD, Queensland; SA, South Australia; TAS, Tasmania; VIC, Victoria; WA, Western Australia.

P1-T1 MRMP was detected in international genomes throughout the phylogeny; however, cases in Australia belonged to clade T1-A (ST3, n = 13/13) exclusively (Figure 2*B*, Figure 3*A* and Figure 3*B*). Interestingly, all clade T1-A cases contained MRMP only, which had not been reported within the Oceania region prior to this study. From the historical cohort we detected a single MRMP genome (3%, 1/31); this specimen was collected in 2018 and also phylogenetically grouped in clade T1-A, ST3 (Figure 2 and Supplementary Data 2). T1-C, the most common *M. pneumoniae* clade in this study (27%, 33/124), mainly represented by ST3, did not contain MRMP mutations.

P1-T2 MRMP genomes were restricted to clade T2-H (n = 3/19), with 1 case reporting recent travel history to China. In our study, the only macrolide resistance-associated mutation identified was the 23S rRNA A2063G substitution. This mutation was detected in all clade T1-A and 3 clade T2-H genomes (13%, 16/124) (Figure 3*A*).

Resistance was found across large geographic regions of Australia (Figure 3*F* and Supplementary Data 3, Supplementary Figure 3). No additional resistance-conferring mutations for tetracyclines or quinolones were detected.

### Assessment of Health Care Utilization

Health care admission status was not significantly associated with genomic-based MRMP detection (*P* = .3351) but was significantly associated with MRMP PCR detection (*P* = .018). The odds ratio (OR) for health care admission was significantly lower for T1-B (OR, 0.092; 95% CI, .011–.54; *P* = .0142) and T2-G (OR, 0.20; 95% CI, .04–.91; *P* = .0426), and both clades did not contain MRMP cases (Figure 3*C*, *D* and *E* and Supplementary Data 3, Supplementary Figure 4).

### Assessment of Low-Diversity Clusters

Phylogenetic analysis also highlighted several clusters that represent genomes with limited genetic diversity (< 10 SNPs), collected during the 2023–2024 outbreak, specifically T1-D (n = 13/13, 0–5 SNPs) and T2-G (n = 16/18, 0–5 SNPs; Supplementary Data 3, Supplementary Figure 5). These clusters may have represented transmission events; however, they had significant temporal and/or geographical distribution. Both clusters contained cases collected from at least 4 Australian states and represented specimens collected between 0 and 30 weeks apart.

### Time-Resolved Phylogenetic Analysis

Time-resolved phylogenies suggest the clades detected in Australia have long evolutionary histories, which are not well represented by publicly available *M. pneumoniae* genomes (Supplementary Data 3, Supplementary Figures 6 and 7). Circulation of genomes in clade T1-F (ST17, n = 14) have evolutionary histories linked to ST1/ST2 strains, which diversified in the early 1900s. More recently, in the early 1950s, *M. pneumoniae* strains diversified into 3 distinct phylogenetic clades, here labeled T1-A, T1-B and C, and T1-D and E. The diversification and expansion of clade T1-A (mainly ST3) occurred in the early 1990s, where all strains contain mutations conferring resistance to macrolides. In contrast, time-resolved phylogenetic analysis of P1-T2 strains demonstrates bifurcation of T2-G (mainly ST7) and T2-H (mainly ST14), predicted to have occurred between 1765 to 1845. T2-G likely emerged in the late 1800s whereas T2-H is predicted to have emerged between 1945 and 1970. MRMP cases have been reported in both clades, initially in T2-H during the early 2000s, followed by a recent expansion of MRMP genomes in clade T2-G around 2015.

### Macrolide-Resistant RT-PCR Detection

To support tNGS analysis, macrolide resistance was also detected using RT-PCR. In this study, MRMP was detected in 12% (43/350) of specimens using RT-PCR (2% [6/356] failed to amplify) (Figure 3*F*). MRMP was also detected in the historical cohort of specimens (13%, 4/31, 2014–2021) with the earliest MRMP cases identified in 2016, followed by a single case in 2018 and 2 cases in 2019. Macrolide resistance was observed in multiple Australian jurisdictions. However, RT-PCR failed to detect 1 MRMP that was identified by tNGS. (Figure 3*F* and Supplementary Data 3, Supplementary Figure 8).

## DISCUSSION

This study demonstrated the capacity of tNGS to assess the genetic diversity of *M. pneumoniae* and identify mutations conferring antibiotic resistance directly from clinical specimens. Our findings provide further support for the use of tNGS in genomic epidemiology investigations for pathogens that are difficult to culture or where PCR has replaced bacterial culture, thereby limiting culture-based whole-genome sequencing or phenotypic antimicrobial susceptibility testing [40, 41].

Macrolides remain the first-line treatment for *M. pneumoniae*; this study provides strong evidence supporting the continued use of macrolides in Australia [2]. However, our findings also highlight the need for close monitoring of MRMP and MRMP testing in cases of suspected treatment failure. For over 25 years, studies from Asia have reported periodic increases in MRMP, with recent postpandemic reports showing nearly universal macrolide resistance [7, 29–32, 42, 43]. This contrasts with low MRMP rates in Europe and the United States (eg, < 2% in Denmark, 3% Germany, 3% The Netherlands, 4.5% United States) [11, 12, 44, 45].

Traditional typing has focused on the P1 adhesin protein, which enables *M. pneumoniae* to adhere to host cells. Shifts in dominant P1 types have been proposed to drive new epidemic waves as population immunity wanes [30]. During the 2023–2024 epidemic in Australia, both P1 types (69% P1-T1 and 31% P1-T2) circulated, similar to China, where the dominant strain was P1-T1 MRMP (ST3; T1-A in this study), and approximately 30% of cases were P1-T2 (ST14; T2-G in this study); in both P1 types almost all cases were MRMP.

A small number of genomic studies have suggested that MRMP may be driven by clonal expansion of specific phylogenetic clades [7, 8]. We identified low (13%) but increasing MRMP rates across Australia during the 2023–2024 epidemic [20]. Historical Australian studies conducted in 2014 and 2016 reported 3.3% and 0% of *M. pneumoniae* cases were resistant to macrolides [25, 26].

All Australian P1-T1 MRMP cases belonged to T1-A and clustered with ST3 genomes, a lineage that, until the 2024 outbreak, had only been reported in Asia but has subsequently been detected in *M. pneumoniae* studies in the United Kingdom from 2024 [46]. However, the majority of Australian P1-T1 cases were distributed across several underrepresented macrolide-sensitive clades, highlighting a diverse and undersampled *M. pneumoniae* population largely unrecognized outside Asia.

MRMP was also detected in Australian P1-T2 genomes (3/19 cases). Unlike China, both major ST14 clades circulated, including susceptible strains. One MRMP ST14 case was linked to travel from China, but genomic evidence indicated local transmission, with cases detected in multiple states over 3 months. Four historical MRMP cases from 2016–2019 (4/31) provide further evidence for rising resistance in Australia [25, 26]. The geographical variation of MRMP detection in our study across Australia states (0%–29% by PCR) likely reflects different importation rates, referral sources, and sampling biases.

Globally, genomic data show that MRMP has emerged in nearly all *M. pneumoniae* phylogenetic clades. Because resistance requires only a single-point 23S rRNA mutation, the barrier to resistance is low. However, clade T1-A in this study consisted entirely of MRMP genomes, suggesting clonal expansion. Time-resolved phylogenetic analysis supports rapid expansion of T1-A beginning in the late 1990s, coinciding with the first clinical MRMP reports in 2000. This expansion may reflect selective pressures from increased or excessive macrolide use [47] for nonrespiratory infections, and/or increased virulence or fitness of this clade [38].

Understanding the selective landscape shaping MRMP emergence is critical to predicting how rapidly MRMP could expand in countries with low prevalence. This is particularly relevant for antimicrobial stewardship and, notably, macrolides are also first-line treatments for other common bacterial infections such as *Chlamydia trachomatis* and *Bordetella pertussis*, where macrolide resistance is also on the rise [48–50].

Although *M. pneumoniae* infection is often mild and self-limiting, 42% of our cohort were tested for *M. pneumoniae* whilst attending a health care facility, and 32% required hospital admission. The convenience sampling used in our study may overestimate health care burden. This interpretation is supported by Danish national surveillance data demonstrating no increased hospitalization risk during the 2023–2024 epidemic; however, the MRMP prevalence was only 1.5% in this cohort. In contrast, we observed that MRMP cases were significantly more likely to seek health care (*P* = .018). In addition, a lower age-adjusted OR of health care utilization was associated with macrolide-sensitive clades (T1-B, ST3, n = 33 and T2-G, ST7, n = 20), compared with MRMP clade T1-A. These findings suggest an association between MRMP and increased health care utilization, likely reflecting either treatment failure or confounding by indication, whereby more severe disease is both more likely to be treated and resistant. Some other limitations of the current study are acknowledged, including the dependence of tNGS success on *M. pneumoniae* load in clinical specimens potentially biasing our cohort toward more severe cases or prolonged infections. The relatively small study cohort could also result in missing other macrolide resistance mechanisms (23S rRNA mutations at 2353 and 2617). It is also acknowledged that while tNGS provided high-resolution genomic context, the RT-PCR assay demonstrated superior sensitivity for macrolide resistance detection specifically, with results available across a larger proportion of the cohort (350 vs 124 specimens). As all MRMP cases identified by tNGS in this study harbored the A2063G mutation, which is directly targeted by the RT-PCR assay, the latter represents a more scalable and sensitive tool for routine MRMP surveillance, albeit missing 1 MRMP case that was identified by tNGS. tNGS remains indispensable for characterizing genomic diversity, identifying emerging resistance mechanisms, and enabling phylogeographic inference, functions that PCR alone cannot provide. Future surveillance would benefit from deploying both approaches in a tiered strategy; RT-PCR for high-throughput resistance screening, with tNGS applied to representative samples or cases of suspected treatment failure.

In conclusion, targeted metagenomic sequencing enabled genomic surveillance of *M. pneumoniae*, revealing a diverse macrolide-sensitive population with merging macrolide resistance driven partly by clonal expansion of MRMP clades. The cocirculation of multiple P1 adhesin types, including macrolide-susceptible and -resistant strains, underscores the dynamic nature of *M. pneumoniae* epidemiology. Importantly, the association between MRMP and increased health care utilization suggests that resistance has clinical consequences. These results highlight the need for continued genomic surveillance and antimicrobial stewardship to guide effective treatment strategies and mitigate the potential spread of resistant strains in future epidemics.

## Supplementary Material

jiag163_Supplementary_Data

## Supplementary Data

Supplementary materials are available at *The Journal of Infectious Diseases* online (http://jid.oxfordjournals.org/). Supplementary materials consist of data provided by the author that are published to benefit the reader. The posted materials are not copyedited. The contents of all supplementary data are the sole responsibility of the authors. Questions or messages regarding errors should be addressed to the author.
