# Supplementary material for: Genomic Surveillance Reveals Emergence and Spread of Macrolide-Resistant Mycoplasma pneumoniae in Australia During the 2023–2024 Epidemic
Source: J Infect Dis. 2026 Mar 21;234(1):e121–30. doi: 10.1093/infdis/jiag163 (PMC13431891; doi:10.1093/infdis/jiag163)
Supplement: jiag163_Supplementary_Data [file jiag163_supplementary_data.zip › Supplementary data 3 _JID_no_markup.pdf]

Supplementary Data 3

Genomic Surveillance Reveals Emergence and Spread of Macrolide-Resistant *Mycoplasma pneumoniae* in Australia During the 2023–2024 Epidemic

Kingsley King-Gee Tam[1,2], Carl J E Suster[1,2], Winkie Fong[1,2], Tanya Golubchik[1,2], Varsha Sivalingam[1,3], Neisha Jeoffreys[3], Enoch Tay[3], Danny Ko[3], Michael C Wehrhahn[4], Andrew N Ginn[4], Jennifer Robson[5], Indya Gardner[5], Lito E Papanicolas[6], Karina Kennedy[7], Maryza Graham[8,9,10,11,12], Thomas Tran[8], David Speers[13], Louise Cooley[14], Rob W Baird[15], Ella M Meumann[1516] Jaimee Harbidge[15], Stuart Campbell[15], Kerri Basile[1,2,3], Sharon C-A Chen[1,2,3] Vitali Sintchenko[1,2,3], Jen Kok*[1,2,3] and Rebecca J Rockett*[1,2]

1. Centre for Infectious Diseases and Microbiology – Public Health, Westmead Hospital, Westmead, New South Wales, 2145, Australia
2. Sydney Infectious Diseases Institute, The University of Sydney, Camperdown, New South Wales 2006, Australia
3. Centre for Infectious Diseases and Microbiology Laboratory Services, NSW Health Pathology - Institute of Clinical Pathology and Medical Research, Westmead Hospital, Westmead, New South Wales 2145, Australia
4. Douglass Hanly Moir Pathology, A Sonic Healthcare Practice, Macquarie Park, New South Wales, 2113, Australia
5. Sullivan Nicolaides Pathology, A Sonic Healthcare Practice, Bowen Hills, Queensland, 4006, Australia
6. SA Pathology, Adelaide, South Australia, 5000, Australia
7. ACT Pathology, Woden, Australian Capital Territory, 2606, Australia
8. Victorian Infectious Diseases Reference Laboratory, Royal Melbourne Hospital, Doherty Institute for Infection and Immunity, Melbourne, Victoria, 3000, Australia
The Victorian Infectious Diseases Reference Laboratory (VIDRL), Melbourne, Victoria, 3000, Australia
9. Department of Infectious Diseases, University of Melbourne, Doherty Institute for Infection and Immunity, Melbourne, Victoria, 3000, Australia
10. Department of Microbiology, Monash Health, Clayton, Victoria, 3800, Australia
11. Monash Infectious Diseases, Monash Health, Clayton, Victoria, 3800, Australia
12. Faculty of Medicine, Nursing and Health Sciences, Monash University, Clayton, Victoria, 3800, Australia
13. PathWest Laboratory Medicine WA, Department of Microbiology, Nedlands, Western Australia, 6006 Australia
14. Department of Microbiology and Infectious Diseases, Royal Hobart Hospital, Hobart, Tasmania, Australia
15. Territory Pathology, Royal Darwin Hospital, Casuarina, Northern Territory, 0811, Australia
16. Global and Tropical Health Division, Menzies School of Health Research, Casuarina, Northern Territory, 0811, Australia

* These authors contributed to this work equally.
Corresponding author: Rebecca J Rockett Rebecca.rockett@sydney.edu.au

Table of Contents

SUPPLEMENTARY DATA 3 1

GENOMIC SURVEILLANCE REVEALS EMERGENCE AND SPREAD OF MACROLIDE-RESISTANT *MYCOPLASMA PNEUMONIAE* IN AUSTRALIA DURING THE 2023–2024 EPIDEMIC 1

SUPPLEMENTARY METHODS 3
SUPPLEMENTARY RESULTS 5
FIGURE S1 *M. PNEUMONIAE* PATHOGEN LOAD AND DNA EXTRACT QUALITY ARE PREDICTORS OF SUCCESSFUL TNGS GENOME RECOVERY 8
FIGURE S2. SUMMARY OF GENOME-WIDE READ COVERAGE GENERATED BY TNGS FOR P1 ADHESION TYPE 1 & 2 *M. PNEUMONIAE* REFERENCE GENOMES 9
FIGURE S3. DIFFERENT RATES OF MACROLIDE RESISTANT *M. PNEUMONIAE* DETECTED IN AUSTRALIAN STATES AND TERRITORIES 10
FIGURE S4. AGE ADJUSTED ODDS RATIOS TO ACCESS RISK OF HEALTHCARE OF EACH PHYLOGENETIC *M. PNEUMONIAE* CLADE. 11
FIGURE S5. GENOME WIDE SNP DIFFERENCES BETWEEN PHYLOGENETIC CLADES OF *M. PNEUMONIAE* IN AUSTRALIA 12
FIGURE S6. TIME RESOLVED PHYLOGENETIC ANALYSIS OF *M. PNEUMONIAE* P1 ADHESION TYPE 1 14
FIGURE S7. TIME RESOLVED PHYLOGENETIC ANALYSIS OF *M. PNEUMONIAE* P1 ADHESION TYPE 2 16
FIGURE S8. OPTIMISATION OF PCR CYCLE THRESHOLD DIFFERENCE THAT ACCURATELY DETECT MRMP CASES 18
REFERENCES 19

Supplementary Methods

MP is not a notifiable infectious disease in Australia therefore monitoring the size and breadth of epidemics is difficult. To quantify the surge in MP cases in Australia in 2024 weekly MP polymerase chain reaction (PCR) positivity data was supplied by two large private pathology providers, Douglass Hanly Moir Pathology and Sullivan Nicolaides Pathology (Sonic HealthCare Limited, Australia) between 1st January 2018 – 7th October 2024. These service general practitioners and specialist medical practitioners across urban and rural New South Wales (NSW) and Queensland (QLD), respectively.

Sample cohort

A total of 356 MP PCR positive specimens, collected from 352 patients were referred from public (SA Pathology (South Australia (SA), n=47), Territory Pathology (Northern Territory (NT), n=34), ACT Pathology (Australian Capital Territory (ACT), n=58), The Victorian Infectious Diseases Reference Laboratory (VIDRL, Victoria (VIC), n=41), PathWest Laboratory Medicine (Western Australia (WA), n=27), Royal Hobart Hospital (Tasmania, (TAS) n=2), NSW Health Pathology- Institute of Clinical Pathology and Medical Research (ICPMR, NSW, n=19); and private (Douglass Hanly Moir Pathology (NSW, n=49), Sullivan Nicolaides Pathology (QLD, n=79)) diagnostic laboratories in all jurisdictions of Australia.
These included MP specimens that were referred to the Centre for Infectious Diseases and Microbiology – Public Health. Respiratory tract specimens collected included nasopharyngeal aspirates (35%, 123/356), nose and throat swabs (21%, 73/356), sputum (18%, 64/356), throat swabs (8%, 28/356), nose swabs (2%, 7/356), lavage (2%, 8/356), plural fluid (0.8%, 3/356), bronchial wash (0.2%,1/356) and a tracheal aspirate (0.2%,1/356): the swab site was unspecified in 13% (48/356) cases. Where the data was available a history of overseas travel and antibiotic treatment in the two weeks prior to sample collection was captured.

Library preparation

Total nucleic acid was extracted at the referring laboratory or prior to tNGS library preparation at ICPMR using the MagNA Pure 96 DNA and Viral NA Small Volume kit (Roche Life Science, Basel, Switzerland) on the MagNA Pure 96 instrument (Roche Life Science, Basel, Switzerland) with a final elution volume of 100ml. Libraries were prepared using the Enzymatic Fragmentation (EF) kit 1.0 (Twist Biosciences, South San Francisco, USA). The quantity of tNGS libraries was assessed using the Qubit dsDNA High Sensitivity Quantitation Assay (Thermo Fisher Scientific, Massachusetts, USA). An equal volume pool containing 2mL of each tNGS library was prepared from up to 48 libraries and DNA was dried down prior to overnight (17 hour) hybridisation using the TWIST Target Enrichment Standard Hybridization. Hybridisation using two bait panels was conducted for each library pool. A custom bait panel, was designed using the core and accessory genome of 474 M. pneumoniae reference genomes. This design was generated to capture the entire MP pangenome (MP panel) and consisted of 13936 120-mer oligonucleotides probes (panel design available on request). The libraries were co-captured with the Castanet probe panel, which has been previously described[1] The *National Center for Biotechnology Information* (NCBI) Genbank accessions of MP genomes used for panel construction are listed in Supplementary Data 1. Post-capture libraries were quantified using an Agilent Bioanalyzer High Sensitivity DNA Kit (D5000) (Agilent Technologies, California, USA) and a Thermo Fisher Scientific Qubit dsDNA High Sensitivity Quantitation Assay to calculate the molarity of each post-capture library pool.

Samples were sequenced using Illumina chemistry resulting in 150 bp paired-end reads with the aim of generating 1-2 million reads per sample.

Bioinformatic analysis

Sequencing reads were trimmed to remove Illumina adapter sequences and poor-quality reads using Trimmomatic. [2] Demultiplexed sequence read pairs were classified by Kraken2. [3] To place Australian MP genomes into the global context a collection of raw reads and reference genomes were downloaded from NCBI (n=474) and included in further analysis (Supplementary Data 1). Trimmed reads were mapped to the chromosome of the NCBI RefSeq assembly CP003913.2 using Snippy (version 4.6.0). [4] Reference-based mapping quality was assessed using samtools (version 1.10) and only MP genomes with >80% genome coverage and a minimum of 12x depth were included for further analysis. A full genome alignment was generated using Snippycore (version 4.1.0) and recombination was removed using Gubbins (version 2.3.4). [5] Single nucleotide polymorphism (SNP) sites were used to determine core SNP differences between genomes. [6] Maximum-likelihood phylogenetic reconstruction was performed on the recombination-masked SNP alignment with automated model selection as implemented in IQ-TREE [7] (version 1.6.7) and 1000 bootstrap replicates with zero-length branches collapsed. Bifurcation of the phylogeny demonstrated deep divergence, clearly separating genomes into clusters of P1-T1 and P1-T2. To enhance visualisation of closely related genomes, reads were remapped based on the P1 adhesin type to either a P1-T1 (CP003913.2) or P1-T2 (NZ_LR214945.1) reference genomes. A phylogeny was then reconstructed for each P1 adhesin type as outlined above.

To estimate the timing of divergence events, we applied BactDating (version 1.1), a Bayesian framework for dating bacterial phylogenies using tip dates. [8] The midpoint-rooted phylogeny constructed for both P1-T1 and P1-T2 was used with branch lengths rescaled to represent the number of substitutions by multiplying by the alignment length (3484 bp P1-T1 and 1848 bp P1-T2).

Macrolide resistance was detected *in silico* from variant calling at 23S rRNA positions on the consensus genomes. In addition, to limit potential mis-mapping from 23S rRNA reads from other bacterial species[9], trimmed reads were psuedomapped to the SILA 23S rRNA database using kallisto (version 0.46.0). [10] Reads mapping specifically to the *M. pneumoniae* 23S rRNA reference sequences were then extracted and mapped to the 23S rRNA NCBI RefSeq assembly GCF_900660465.1 using bwa-MEM. [11] Consensus genomes were then aligned and mutations at nucleotide positions 2017, 2054, 2058, 2063, 2064, 2071, 2353 and 2617 were investigated. [12,13]

Additional resistance markers were investigated including tetracycline resistance mutations in the 16S rRNA (G1193A, T968C), quinolone resistance by mutations in *gyrA*, *gyrB*, and *parC.* [12]

Macrolide resistance by RT-PCR

All clinical specimens were subjected to a modified MP 23S rRNA resistance real-time PCR assay [14] that was performed in a 25 mL reaction using the Rotor-Gene Q 5plex HRM platform (Qiagen, Hilden, Germany). Each reaction consisted of 1x SensiFAST Probe No-ROX mix (Bioline, London, UK.), 0.5 mM of each forward and reverse primer, 0.3 µM of MP 2063/2064 probe, 0.1 mM of MP 23S probe and 3 mL of DNA as the template. The PCR was carried out under the following conditions: initial denaturation at 95°C for 5 min, followed by 45 cycles of amplification at 95°C for 10 seconds and 60°C for 30 seconds. Fluorescent signal acquisition was conducted at the end of every cycle during the amplification stage and cycle threshold (Ct) was determined by Rotor-Gene Q (Qiagen, Hilden, Germany) software

for each reaction. Specimens with DCt (Ct_{YAK} – Ct_{FAM}) that exceeded 1.5 were interpreted as MRMP.

Statistical analysis

Genome read depth and genome coverage were compared between freshly re-extracted original samples and referred DNA extracts using a Kruskal-Wallis rank sum test. We divided healthcare access into two categorical variables, limited (community) or hospital attendance (ED, inpatient and ICU). To assess statistical association between MRMP and healthcare access, a Pearson's Chi-squared test with Yates' continuity correction was performed to assess if healthcare access was statistically different between MRMP and macrolide susceptible MP cases. To assess the association between phylogenetic group and healthcare access an age-adjusted logistic regression was used. The primary predictor was clade (categorical), with the reference group set to the T1-A clade (n=13). Odds ratios (ORs) and 95% confidence intervals (CIs) were estimated for each clade relative to the reference. T1-C (n=3) was excluded from the analysis due to small sample size to improve model reliability. Statistical significance was defined as a 95% CI for the odds ratio that did not include 1. Statistical analysis and visualisation were performed using R (version 4.2.2).

Supplementary Results

Resurgence of M. pneumoniae in Australia in 2023 and 2024

MP PCR positivity between 2018 - 2022 was less than 10% (Figure 1A). However, from late 2023 (epidemiological week 47) PCR positivity surpassed 10% reaching 21% by the first week of 2024 and peaking at 31% by week 14, 2024. MP detection rate remained >10% until mid 2024 (week 27 2024).

Demographics of the cohort indicated 44% (157/357) of MP cases were diagnosed in children less than 15 years of age (Figure 1B & C). Just over half of MP cases (52%, 186/352) in the study were managed in the community, however 44% required hospital presentation, comprising ED presentation 10% (35/352), hospital admission 31% (109/352) and ICU admission 3% (10/352). Healthcare seeking behaviour was not available for 5% (17/352) of MP cases (Figure 1B). As no epidemiological follow-up was conducted, travel history within 14 days of sample collection and antibiotic treatment history were available for only 4/352 and 6/352 MP detections respectively.

Genomic analysis

tNGS was attempted on 322 specimens, this resulted in the recovery of 124 complete MP genomes using tNGS (40%, 124/322) (Figure 2A). Of the complete genomes 15 cases were from the historical MP cohort (collected between 2014 – 2021) and 109 from the recent epidemic (2023-2024). Successful genome recovery was associated with lower MP PCR Ct values, where specimens with Ct ≤20 had a whole MP genome recovery rate of 94% (16/17). Despite whole genomes being successfully recovered in MP specimens with low bacterial load (Highest Ct value: 32.07), a lower genome recovery rates were observed with increasing Ct values ranges, 20 -<25 (53%), 25-<30 (28%) and 30-<35 (14%) (Supplementary Data 3, Figure S1). It was also noted that when original specimens were re-extracted prior to tNGS these specimens yielded a significantly higher genome recovery rate, read depth and genome

coverage when compared to DNA samples transported from other sites across all but one Ct range (read depth; p-value = 0.003, genome coverage p-value = 0.023) (Supplementary Data 3B, Figure S1). High quality genomes were recovered with an average depth of 121.5x (range 13 – 2579x) and genome coverage of 99.81% (range 99.2 - 100) (Supplementary Data 2 and Supplementary Data 3, Figure S2).

The MP phylogeny was constructed by including 474 international MP genomes (Supplementary Data 1). The majority of available genomes were collected from Asia (86%, n=407) with much lower geographical representation of MP genomes from Europe (5%, 26/474), North America (7%, 32/474) South America (0.4%, 2/474), the Middle East (0.4%, 2/474) and Africa (1.1%, 5/474).

The MP phylogeny demonstrated deep divergence into two primary branches, containing study genomes classified as P1-T1 (69% (85/124)) or P1-T2 (31% (39/124)). Both P1-adhesin types co-circulated during 2023-2024, however P1-T1 dominated (Figure 2A). Due to the deep divergence, a separate phylogenetic analysis was conducted for each P1 type. The P1-T1 genomes produced in this study, had a high degree of genetic diversity falling into six of nine major clades: T1-A (n=13), T1-B (n=3), T1-C (n=33), T1-D (n=13), T1-E (n=9) and T1-F (n=14) (Figure 2B). MLST was inferred from the consensus genomes and supported the phylogenetic classifications, however whole genome analysis further differentiated ST3 (clades T1-A and T1-B) and ST20 (clades T1-D and T1-E).

Genomics-based resistance assessment

P1-T1 MRMP was detected in international genomes throughout the phylogeny, however cases in Australia belonged to Clade T1-A (ST3, n=13/13) exclusively. Interestingly, all Clade T1-A cases contained MRMP only, all of which prior to this study, were reported from countries within the Asian region. In our cohort only a small set of MP positive specimens had been archived and were available for our study, and from this historical cohort we detected a single MRMP genome (3%, 1/31), this specimen was collected in 2018 that also phylogenetically grouped in Clade T1-A, ST3 (Figure 2, Supplementary Data 2). T1-C, the most common MP clade in this study (27%, 33/124) mainly represented by ST3, did not contain MRMP mutations.

Phylogenetic analysis of P1-T2 genomes demonstrated that Australian cases were clustered into two of four major clades: T2-G (ST7, n=20) and T2-H (ST14, n=19) (Figure 2C). P1-T2 MRMP genomes were restricted to clade T2-H (n=3/19), with one case reporting recent travel history to China. In our study the 23S rRNA mutation A2063G was detected in all Clade T1-A and three clade T2-H genomes (13% 16/124).

Resistance was found across large geographic regions of Australia with MRMP cases detected in NSW (17%, n=8/46, Clade T1-A n=6, Clade T2-H, n=2), VIC (15%, n=3/20, Clade T1-A), NT (9%, n=1/11, Clade T1-A), SA (16%, n=1/6, Clade T1-A), ACT (14%, n=1/7, Clade T1-A) and WA (17%, n=2/12, Clade T1-A n=1, Clade T2-H n=1) (Figure 3A, 3F and Supplementary Data 3, Figure S3).

No additional resistance conferring mutations for tetracyclines or quinolones were detected.

Assessment of healthcare utilisation

Healthcare admission status was not significantly associated with genomic based MRMP detection (*p*= 0.3351). The OR for healthcare admission was significantly lower for T1- C (OR

0.092 (95% CI 0.011 0.54) p = 0.0142) and T2-G (OR 0.20 (95% CI 0.04 0.91), p = 0.0426) and both clades did not contain MRMP cases (Figure 3D, Supplementary Data 3 Figure S4).

Assessment of low diversity clusters

Phylogenetic analysis also highlighted several clusters which represent genomes with limited genetic diversity (<10 SNPs), collected during the 2023/2024 outbreak, specifically T1-D (n=13/13, 0-5 SNPs) and T2-G (n=16/18, 0-5 SNPs; Supplementary Data 3 Figure S5). These clusters may have represented transmission events; however, they had significant temporal and/or geographical distribution. Both clusters contained cases collected from at least four Australian states and represented specimens collected between 0-30 weeks apart.

Time-resolved phylogenetic analysis

Time-resolved phylogenies suggest the clades detected in Australia have long evolutionary histories, which are not well represented by publicly available MP genomes (Supplementary Data 3, Figure S6 & S7). Circulation of genomes in clade T1-F (ST17, n=14) have evolutionary histories linked to ST1/ST2 strains which diversified in the early 1900s. More recently in the early 1950s MP strains diversified into three distinct phylogenetic clades, here labelled T1-A, T1-B & C and T1-D & E (Supplementary Data 3, Figure S6). Following this diversification, expansion of clade T1-A (mainly ST3) occurred in the early 1990s where all strains contain mutations conferring resistance to macrolides. This clade has only been detected in Asian countries prior to our study (n= 218 from the Asian region and n= 13 from Australia). In Australia, P1-T1 MRMP genomes were only found in the T1-A clade. However, macrolide susceptible T1-B (mainly ST3), were the most common strain in our study (n=36) followed by T1-D (ST20, n=13) and T1-E (ST20, n=9) (Figure 3A, Supplementary Data 3, Figure S6). In contrast, time-resolved phylogenetic analysis of P1-T2 strains demonstrates bifurcation of T2-G (mainly ST7) and T2-H (mainly ST14), predicted to have occurred between 1765 to 1845 (Supplementary Data 3, Figure S7). T2-G likely emerged in the late 1800s whereas the T2-H is predicted to have emerged between 1945-1970. MRMP cases have been reported in both clades, initially in T2-H during the early 2000s, followed by a recent expansion of MRMP genomes in clade T2-G around 2015. In Australia MRMP cases were restricted to the T2-H clade (ST14, n=3/19), which is reportedly the dominant P1-T2 strain circulating in Asia during the 2023/2024 epidemic

Macrolide resistant RT-PCR detection

To support tNGS analysis, macrolide resistance was also detected using RT-PCR. In this study, we detected MRMP in 12% (43/350) of specimens using RT-PCR, following the exclusion of seven specimens with (2% (7/357) that failed to amplify or with recorded Ct values >35 (Figure 3F). MRMP was also detected in the historical cohort of specimens (13%, 4/31, 2014-2021) with the earliest MRMP cases identified in 2016, then a single case in 2018 and two, in 2019. Healthcare admission status was significantly associated with MRMP PCR detection (p= 0.018). Macrolide resistance was detected in multiple Australian jurisdictions including NSW (17%, n=11/66), QLD (3%, n=2/78), VIC (29%, n=12/41), NT (9%, n=3/34), SA (4%, n=2/48), ACT (15%, n=8/54) and WA (15%, n=4/27) (Figure 3F and Supplementary Data 3, Figure S3). RT-PCR validation was performed to ensure the Delta Ct threshold used to determine MRMP detection was both sensitive and specific; an annealing temperature of 60°C was determined to optimally identify MRMP (Supplementary Data 3, Figure S8).

Figure S1 *M. pneumoniae* pathogen load and DNA extract quality are predictors of successful tNGS genome recovery

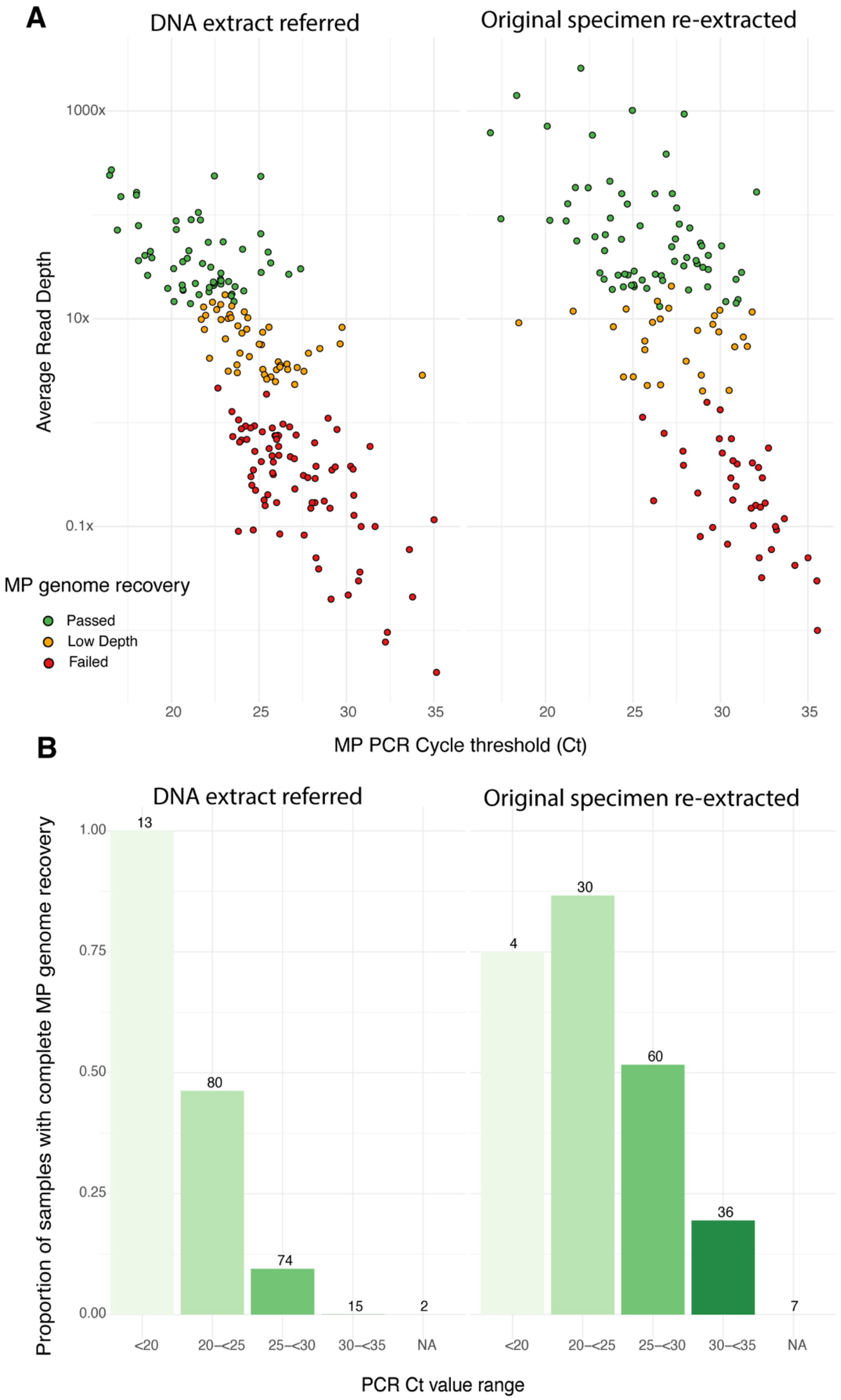

Figure S1. A. Scatter plot to demonstrate the relationship between tNGS *M. pneumoniae* genome read depth and *M. pneumoniae* PCR cycle threshold values. PCR cycle threshold values are semi-quantitative and inversely related to the *M. pneumoniae* DNA concentration in each extract. Green circles indicate successfully recovered *M. pneumoniae* of genomes with a minimum read depth of 12x and percentage genome coverage of 80%, Orange circles indicate genomes with <12x read depth but > 80% genome coverage or cases that failed to meet snippy core quality requirements. Red circles indicate specimens with less than 80% of the *M. pneumoniae* genome recovered at less than 12x read depth. B. Although MP load is predictive of successful genome recovery, DNA quality also contributes to tNGS success. There was a significantly higher rate of complete MP genome recovery from original specimens freshly extracted at the referring laboratory prior to tNGS than specimens referred as DNA extracts (read depth; p-value = 0.003, genome coverage; p-value = 0.023). This indicates DNA extraction quality is likely an important factor in tNGS success. Total numbers of MP samples attempted for each cycle threshold value range are indicated above each bar.

Alt text:
Panel A: Scatter plot and bar chart showing relationship between M. pneumoniae genome read depth by tNGS and PCR cycle threshold values. Green circles indicate successfully M. pneumoniae genomes recovery (≥12× read depth and ≥80% genome coverage), orange circles for genomes with <12× read depth but >80% genome recover, and red circles for genomes with <12× read depth and <80% genome coverage.
Panel B: Bar chart showing higher rates of complete genome recovery from freshly extracted specimens compared with referred DNA extracts, highlighting the impact of DNA quality on tNGS success.

Figure S2. Summary of genome-wide read coverage generated by tNGS for P1 Adhesion Type 1 & 2 *M. pneumoniae* reference genomes

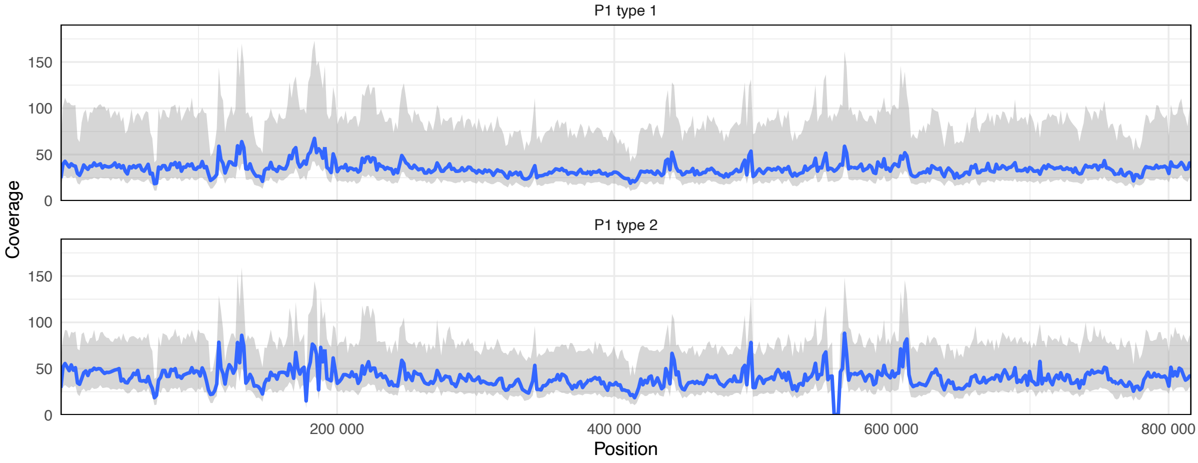

Figure S2. Summary of genome coverage and read depth across *M. pneumoniae* reference genomes P1 Adhesion Type 1 (NCBI accession: CP003913.2) and Type 2 (NCBI accession: NZ_LR214945.1). Blue lines indicate median read depth per genomic position (bin size 1,500 bp), grey lines indicate interquartile range of read depth.

Alt text: Two line plots showing genome-wide read coverage and depth generated by targeted Next-Generation Sequencing (tNGS) for M. pneumoniae P1 Adhesion Type 1 (top) and Type 2 (bottom) reference genomes. The x-axis represents genomic position (0 to 800,000 bp), and the y-axis represents coverage depth.

Blue lines indicate the median read depth per genomic position (1,500 bp bins), while grey shaded areas indicate the interquartile range. Compared with the relatively stable coverage seen across the majority of both genomes, several distinct peaks in depth are observed. Median coverage consistently remains above zero across the entire length of both reference sequences, indicating successful whole-genome enrichment and consistent performance of the sequencing treatment regimen.

Figure S3. Different rates of Macrolide resistant *M. pneumoniae* detected in Australian states and territories

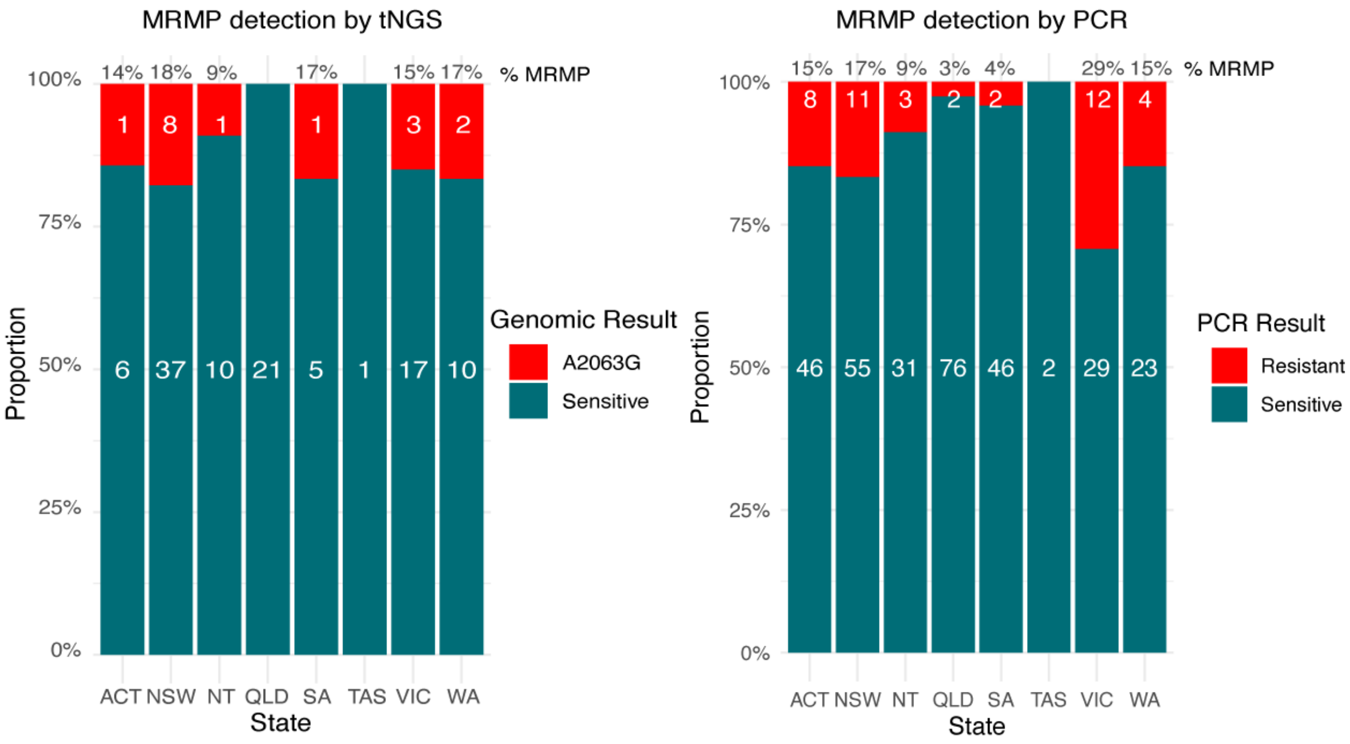

Figure S3. MRMP detection rates across all eight Australian states and territories differed between 0-29%. Low rates (<4%) were detected in Queensland (QLD) and Tasmania (TAS) using both PCR and tNGS detection methods, noting the low number of MP cases from TAS (n=2). Higher rates of MRMP (14 - 29%) where detected in the Australian Capital Territory (ACT), New South Wales (NSW), Victoria (VIC) and Western Australia (WA). MRMP detection between tNGS and PCR was generally concordant, although the PCR method had higher sensitivity and therefore a larger cohort of samples could be analysed. Notable exceptions were the higher rate of MRMP cases detected using PCR (29%) than tNGS (15%) in VIC and lower rates of MRMP detected using PCR (4%) than tNGS (17%).

Alt text: Two stacked bar charts comparing MRMP detection across eight Australian jurisdictions using tNGS (left) and PCR (right) methods. Bars represent the proportion of resistant samples (red, A2063G mutation) versus sensitive samples (green).

Compared with the low-resistance regions of Queensland and Tasmania (0–3%), jurisdictions including ACT, New South Wales, Victoria, and Western Australia showed higher proportions of resistance ranging from 14% to 29%. PCR generally captured a larger sample cohort than tNGS. While results were largely consistent across both methods, the proportion of resistant

cases detected in Victoria was nearly double via PCR (29%) compared to tNGS (15%), whereas in South Australia, the PCR detection rate (4%) was lower than tNGS (17%).

Figure S4. Age adjusted odds ratios to access risk of healthcare of each phylogenetic *M. pneumoniae* clade.

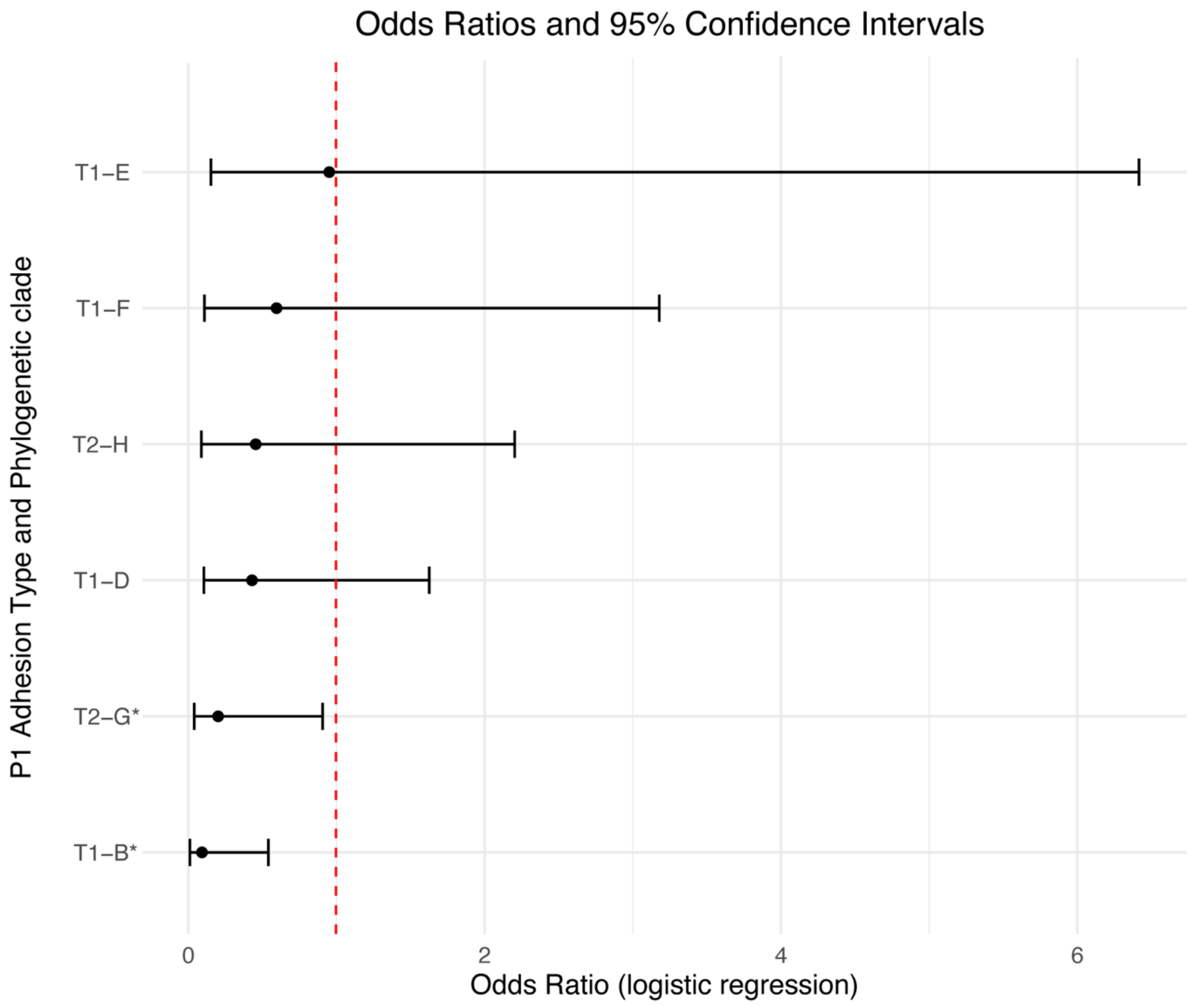

Figure S4. The odds ratio for healthcare admission was significantly lower for MP clades T1-B (OR 0.092 (95% CI 0.011 0.54) p = 0.0142) and T2-G (OR 0.20 (95% CI 0.04 0.91), p = 0.0426) when compared to T1-A. Both clade T1-B and T2-G did not contain genomes with MRMP resistance conferring mutations.

Alt text: Forrest plot showing age-adjusted odds ratios for healthcare admission across six MP phylogenetic clades. Clades T1-B and T2-G, neither of which contained MRMP genomes, had significantly lower odds of healthcare admission compared to the reference

Figure S5. Genome wide SNP differences between phylogenetic clades of *M. pneumoniae* in Australia

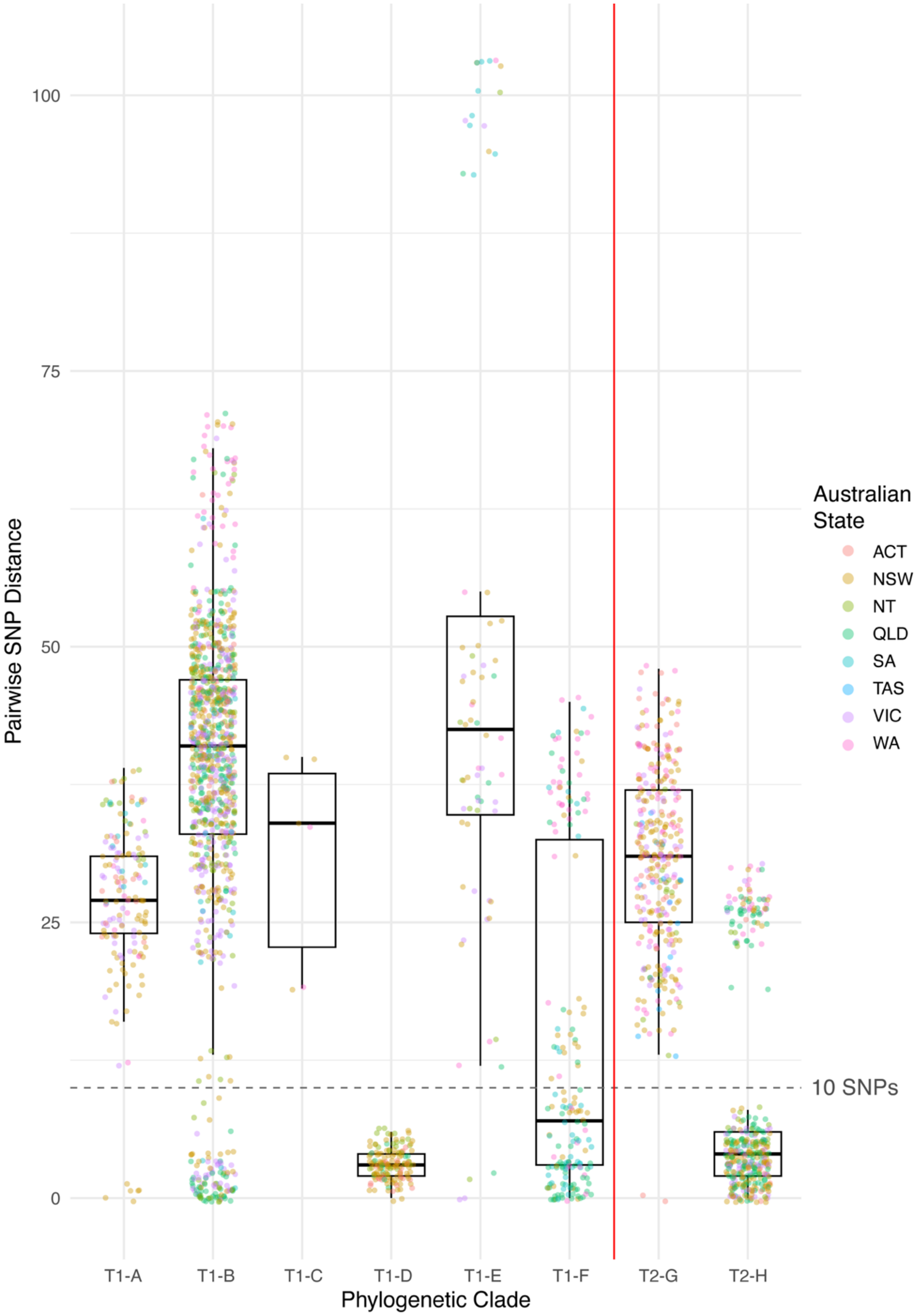

Figure S5. Phylogenetic analysis of whole *M. pneumoniae* genomes revealed clades with limited pairwise SNP distances (≤ 10 SNPs) collected during the 2023/2024 outbreak. This may represent transmission clusters and is particularly noted in Clade T1-D (n=13/13), but

also in subsets of cases in T1-B (n=9/31), T1-F (n=10/12) and T2-H (n=18/20). However, the geographical dispersion of the MP cases, highlighted by the colour jitter in this boxplot suggests co-circulation across multiple Australia states within each of these highly similar clades. Highlighting the circulation of heterogeneous *M. pneumoniae* populations with long evolutionary histories that have been previously under sampled, coupled with the slow evolutionary rate of *M. pneumoniae*.

Alt text: A scatter box plot showing pairwise SNP distances (y-axis) across eight phylogenetic clades of M. pneumoniae (x-axis: T1-A, T1-B, T1-C, T1-D, T1-E, T1-F, T2-G, T2-H), separated into T1 and T2 lineages by a vertical red line. Individual jitters are overlaid on each box and coloured by Australian state (ACT, NSW, NT, QLD, SA, TAS, VIC, WA). A horizontal dashed line marks the 10 SNP threshold. Clades T1-D, T1-F, and T2-H show the majority of points clustered below 10 SNPs, suggesting potential transmission clusters. Points from multiple states appear within each clade, indicating co-circulation of similar strains across Australia. Clades T1-B and T1-E show wider distributions with higher pairwise SNP distances, reflecting greater genomic diversity.

Figure S6. Time resolved phylogenetic analysis of *M. pneumoniae* P1 Adhesion type 1

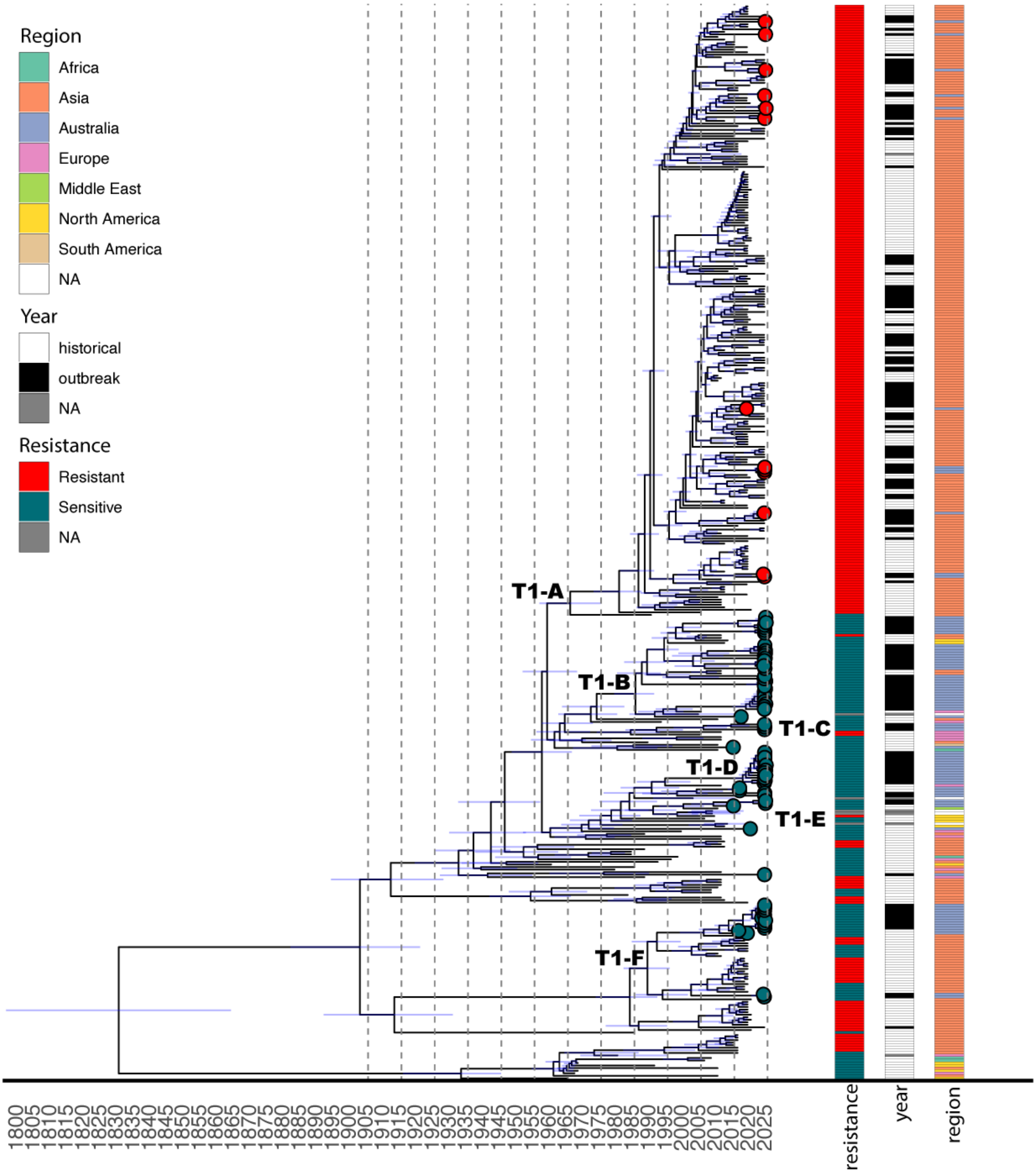

Figure S6. Time resolved phylogenetic analysis of 422 *M. pneumoniae* P1 Adhesion type 1 genomes suggests heterogeneous *M. pneumoniae* strains circulating in Australia. Circulating genomes from clade T1-F (ST17, n=14) have diverged from ST1/ST2 strains in the early 1900s. More recently in the early 1950s MP strains diversified into three distinct phylogenetic clades, here labelled T1-A, T1-B & C and T1-D & E. Following this diversification expansion of clade T1-A (mainly ST3) occurred in the early 1990s and this MRMP clade is reported to dominate Asian *M. pneumoniae* detections. In Australian the T1-A clade contains the only P1-Adhension type 1 MRMP detections. However, macrolide sensitive clades T1-B and T1-C, also mainly ST3 are among the most common strains in our study (n=36) followed by T1-D (ST20, n=13), T1-E (ST20, n=9). Australian genomes (n=85/124) generated for this study are

highlighted by colour circle tips, with Australian genomes containing mutations conferring macrolide resistance highlighted with red circle tips and macrolide sensitive genomes indicated by green tip colour. Confidence internals for predicted node dating demonstrated by blue bars on the tree branches. The left-hand meta bar beside each phylogeny indicates international genomes that contain mutations that confer macrolide resistance which are highlighted in red (n=288) and susceptible genomes in green (n=134). The second metabar indicates MP genomes collected from cases prior to 2023 (n= 256) and attributed to the 2023-2024 epidemic (n=164), year of collection was not available for 2 international genomes. The third metabar indicates the geographical region of collection for each *M. pneumoniae* genome, more than 70% of available genomes are collected from the Asian region (71%, n=301/422) with poor geographical representation of MP genomes collected from Europe (3%, 14/422), North (4%, 15/422) and South America (0.5%, 2/422), the Middle East (0.2%, 1/422) and Africa (1%, 4/422).

Alt text: A time-resolved phylogenetic tree of 422 M. pneumoniae P1 adhesion type 1 genomes, with a timeline along the x-axis spanning from approximately 1800 to 2025. Six clades are labelled on the tree: T1-A, T1-B, T1-C, T1-D, T1-E, and T1-F. Branch lengths represent time, with blue horizontal bars on branches indicating confidence intervals for node dating. Circular tip markers highlight Australian genomes, coloured red for macrolide-resistant and green for macrolide-sensitive strains. Three vertical metadata bars are displayed to the right of the tree. The first bar (resistance) shows each genome coloured red for resistant, teal for sensitive, or grey for not available. The second bar (year) indicates whether genomes were collected historically (white) or during the 2023/2024 outbreak (black), with grey for not available. The third bar (region) shows the geographical origin of each genome using colours corresponding to eight regions: Africa, Asia, Australia, Europe, Middle East, North America, South America, and NA. A legend in the upper left defines the colour coding for region, year, and resistance categories.

Figure S7. Time resolved phylogenetic analysis of *M. pneumoniae* P1 Adhesion type 2

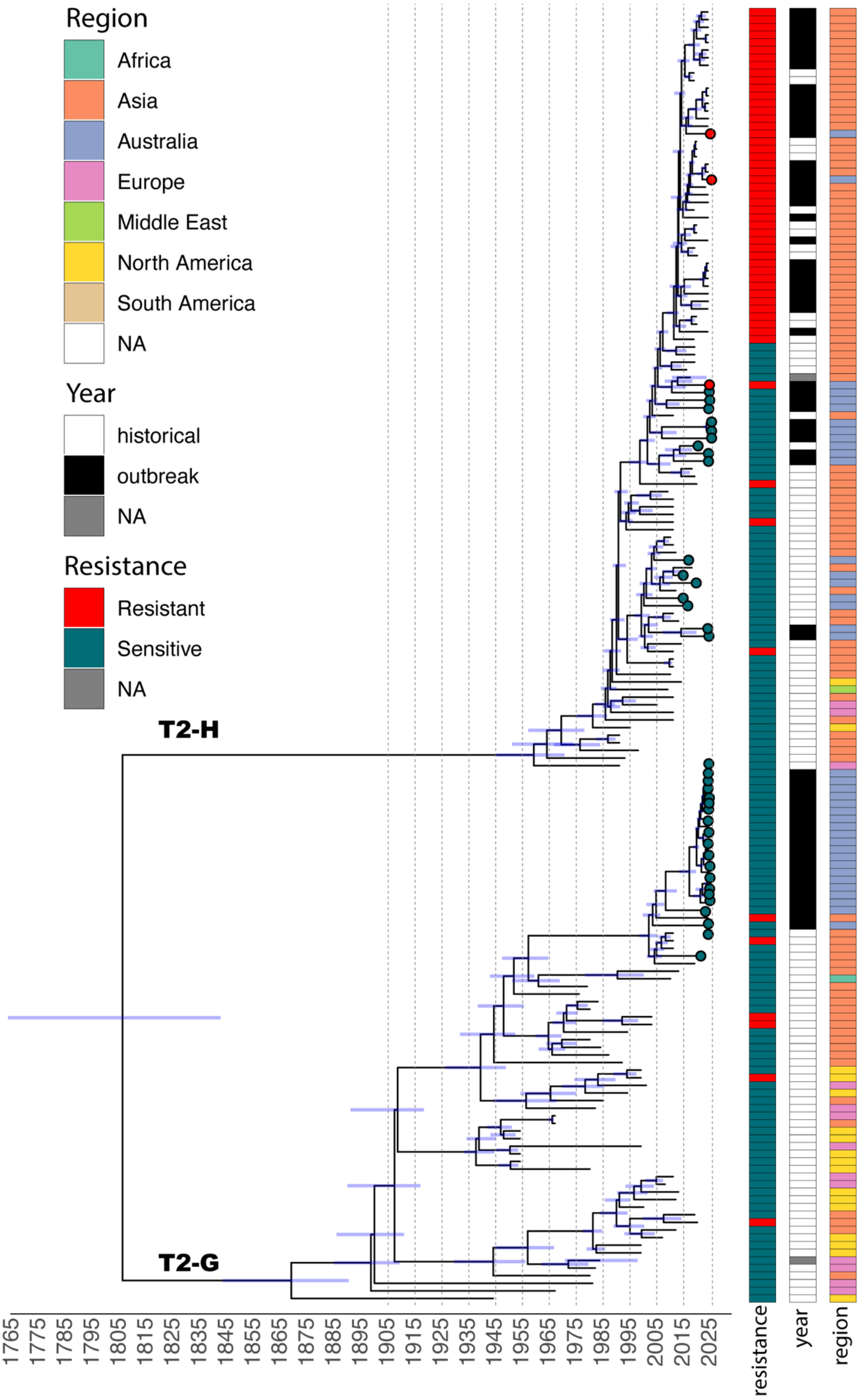

Figure S7. Time resolved phylogenetic analysis of 177 *M. pneumoniae* P1 Adhesion type 2 genomes suggests *M. pneumoniae* strains circulating in Australia (n=39) from both major type 2 clades. Bifurcation of P1 Adhesion type 2 strains was predicted to occur between 1765 to 1845, resulting in the clades labelled T2-G (ST7, n=20) and T2-H (ST14, n=19). The T2-H clade emerged in the late 1800s whereas the T2-G is predicted to have emerged between 1945-1970. MRMP cases have been reported in both clades, initially in the early 2000s in clade T2-H, then a recent expansion of MRMP genomes in clade T2-H around 2015. In Australia MRMP cases were restricted to the T2-H clade, which is reportedly the dominate P1 type 2 strain circulating in Asia during the 2023/2024 epidemic. Australian genomes (n=39/124) generated for this study are highlighted by colour circle tips, with Australian genomes containing mutations conferring macrolide resistance highlighted with red circle tips and macrolide sensitive genomes indicated by green tip colour. Confidence internals for predicted node dating demonstrated by blue bars on the tree branches. The left-hand meta bar beside each phylogeny indicates international genomes that contain mutations that confer macrolide resistance which are highlighted in red (n=55) and susceptible genomes in green (n=122). The second metabar indicates MP genomes collected from cases prior to 2023 (n= 112) and attributed to the 2023-2024 epidemic (n=63), year of collection was not available for 2 international genomes. The third metabar indicates the geographical region of collection for each *M. pneumoniae* genome, more than half of available genomes are collected from the Asian region (60%, n=106/177) with poor geographical representation of MP genomes collected from Europe (7%, 13/177), North America (10%, 17/177) the Middle East (0.6%, 1/177) and Africa (0.6%, 1/177).

Alt text: A time-resolved phylogenetic tree of 177 M. pneumoniae P1 adhesion type 2 genomes, with a timeline along the x-axis spanning from 1765 to 2025. The tree is divided into two labelled clades: T2-H (upper) and T2-G (lower), separated by a common ancestor. Branch lengths represent time, with blue horizontal bars on branches indicating confidence intervals for node dating. Circular tip markers highlight Australian genomes, coloured red for macrolide-resistant and green for macrolide-sensitive strains. Three vertical metadata bars are displayed to the right of the tree. The first bar (resistance) shows each genome coloured red for resistant, teal for sensitive, or grey for not available. The second bar (year) indicates whether genomes were collected historically (white) or during the 2023/2024 outbreak (black), with grey for not available. The third bar (region) shows the geographical origin of each genome using colours corresponding to eight regions: Africa, Asia, Australia, Europe, Middle East, North America, South America, and NA. A legend in the upper left defines the colour coding for region, year, and resistance categories.

Figure S8. Optimisation of PCR cycle threshold difference that accurately detect MRMP cases

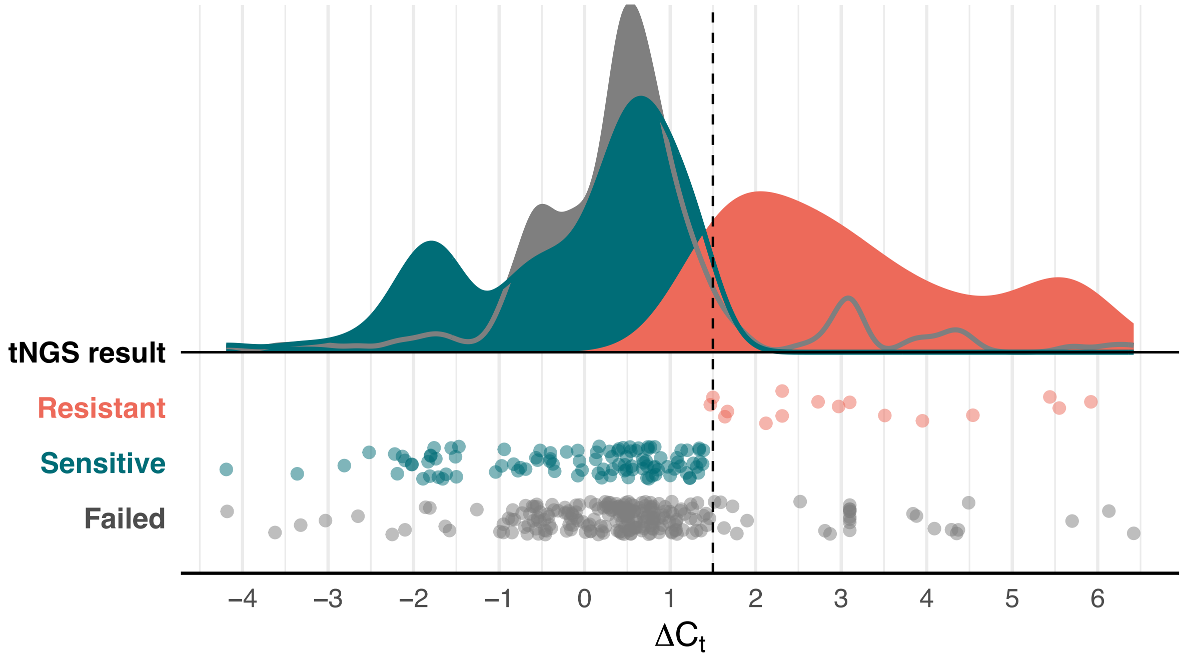

Figure S8. Using MRMP strains in our study detected through tNGS we optimised the PCR conditions to accurate differentiate MRMP cases based on a ΔCT value. We determined a ΔCT of 1.5 optimally identified specimens with resistance conferring mutations. The histogram summaries the frequency of individual cases at each ΔCT, with tNGS confirmed MRMP cases in red and macrolide sensitive cases in green. Specimens that failed tNGS sequencing are coloured in grey with the ΔCT threshold of 1.5 highlighted by the grey dashed line through the graph. A previously published RT-PCR was utilised to detect MRMP conferring mutations at position A2063/4* in the 23s rRNA region, however reagent and equipment modification were required in our study due to reagent unavailability in Australia. Two set of primers were used; one set designed upstream of the region conferring resistance to macrolides that was conserved across all *M. pneumoniae* genomes. The second with a reverse PCR primer contained a A2063A, A2064A base at the 3' end which would most efficiently amplifying sensitive strains. If the infecting strain of *M. pneumoniae* contained a A2063G or A2064G mutation, then the efficiency of amplification would be reduced resulting in a quantifiable difference between the cycle threshold values of the two PCRs, commonly referred to as a ΔCT value.

Alt text: A combined density plot and strip plot, with the x-axis representing ΔCT values. The upper portion shows overlapping density curves for three groups: resistant cases in red, sensitive cases in green, and failed tNGS specimens in grey. The lower portion shows individual ΔCT as a strip plot, with green dots representing tNGS-confirmed macrolide-sensitive cases, red dots representing tNGS-confirmed macrolide-resistant cases, and grey dots representing specimens that failed tNGS sequencing. The vertical dashed line at ΔCT 1.5 spans both the density and strip plot sections, indicating the optimised threshold used to differentiate macrolide-resistant from macrolide-sensitive cases in PCR.

References

1. Lin GL, Drysdale SB, Snape MD, et al. Targeted metagenomics reveals association between severity and pathogen co-detection in infants with respiratory syncytial virus. *Nat Commun* 2024; **15**(1): 2379.
2. Bolger AM, Lohse M, Usadel B. Trimmomatic: a flexible trimmer for Illumina sequence data. *Bioinformatics* 2014; **30**(15): 2114-20.
3. Wood DE, Salzberg SL. Kraken: ultrafast metagenomic sequence classification using exact alignments. *Genome Biol* 2014; **15**(3): R46.
4. Seemann T. Snippy: fast bacterial variant calling from NGS reads. https://github.com/tseemann/snippy: GitHub; 2015.
5. Croucher NJ, Page AJ, Connor TR, et al. Rapid phylogenetic analysis of large samples of recombinant bacterial whole genome sequences using Gubbins. *Nucleic Acids Res* 2015; **43**(3): e15.
6. Page AJ, Taylor B, Delaney AJ, et al. SNP-sites: rapid efficient extraction of SNPs from multi-FASTA alignments. *Microb Genom* 2016; **2**(4): e000056.
7. Nguyen LT, Schmidt HA, von Haeseler A, Minh BQ. IQ-TREE: a fast and effective stochastic algorithm for estimating maximum-likelihood phylogenies. *Mol Biol Evol* 2015; **32**(1): 268-74.
8. Didelot X, Croucher NJ, Bentley SD, Harris SR, Wilson DJ. Bayesian inference of ancestral dates on bacterial phylogenetic trees. *Nucleic Acids Res* 2018; **46**(22): e134.
9. Quast C, Pruesse E, Yilmaz P, et al. The SILVA ribosomal RNA gene database project: improved data processing and web-based tools. *Nucleic Acids Res* 2013; **41**(Database issue): D590-6.
10. Bray NL, Pimentel H, Melsted P, Pachter L. Near-optimal probabilistic RNA-seq quantification. *Nat Biotechnol* 2016; **34**(5): 525-7.
11. Li H, Durbin R. Fast and accurate short read alignment with Burrows-Wheeler transform. *Bioinformatics* 2009; **25**(14): 1754-60.
12. Jiao W, Wang J, Li C, et al. Genetic factors driving the Mycoplasma pneumoniae outbreak among children post-COVID-19 in China: a whole genome analysis. *Lancet Reg Health West Pac* 2025; **59**: 101578.
13. Nguyen DD, Ho NT, Dover LG, et al. Novel Variant and Known Mutation in 23S rRNA Gene of Mycoplasma pneumoniae, Northern Vietnam, 2023. *Emerg Infect Dis* 2024; **30**(5): 1034-6.
14. Liu L, Xiang C, Zhang Y, et al. A Novel Detection Procedure for Mutations in the 23S rRNA Gene of Macrolide-Resistant Mycoplasma pneumoniae with Two Non-Overlapping Probes Amplification Assay. *Microorganisms* 2023; **12**(1).
